# Overview of Torsion Tests on Fiber-Reinforced Concrete Beams

**DOI:** 10.3390/ma18112452

**Published:** 2025-05-23

**Authors:** Jacek Domski, Marek Lehmann, Artur Sanok, Katarina Tvrda

**Affiliations:** 1 Faculty of Civil Engineering, Environmental and Geodetic Sciences, Koszalin University of Technology, 75-453 Koszalin, Poland; marek.lehmann@tu.koszalin.pl (M.L.); artur.sanok@tu.koszalin.pl (A.S.); 2 Faculty of Civil Engineering, Slovak University of Technology in Bratislava, 813 68 Bratislava, Slovakia; katarina.tvrda@stuba.sk

**Keywords:** torsion, load-bearing capacity, cracking stress, beam, steel fibers, database

## Abstract

The article presents the results of laboratory tests published in dozens of publications. These tests focused on reinforced concrete beams with additional dispersed reinforcement that were loaded with torsional moments. The results of cracking and ultimate stress were presented depending on various factors influencing the beam’s load-bearing capacity, i.e., the degree of fiber reinforcement *V_f_*, the angle of rotation *θ*, the compressive strength of the concrete *f_c_*, the fiber slenderness *l/d*, the degree of conventional reinforcement *ρ_tot_*, the proportions of the cross-section *h/b*, and the slenderness of the tested element. The frequency of occurrence of the aforementioned factors in the tests was also statistically analyzed. Additionally, the influence of fibers on the factors determining the torsional load-bearing capacity was examined. It has been shown that fibers can significantly contribute to increasing the torsional load-bearing capacity of beams and can even be characterized by a twofold increase. It has also been observed that despite the increased load-bearing capacity, beams with fibers are often characterized by lower torsional stiffness. Additionally, it has been found that fibers provide the greatest increase in load-bearing capacity for concretes with compressive strengths in the range of 25–45 MPa and for square cross-sections (*b/h* = 1.0). In addition, fibers can lead to the formation of torsional cracks at higher reinforcement factors (*V_f_*). However, in terms of cooperation with conventional reinforcement, fibers are of little importance in transferring torsional stresses in the case of beams with a high conventional reinforcement factor *ρ_tot_* > 3%.

## 1. Introduction

Torsion is an important issue in structural mechanics, influencing the design and analysis of various building elements. A proper understanding of the phenomenon of torsion is essential not only for ensuring the safety and durability of structures but also for the efficient use of building materials. This phenomenon occurs wherever loads do not act in a homogeneous manner. In the context of structures, torsion can be caused by various factors, including asymmetric load distribution, wind forces, and temperature changes. The issue becomes even more complex with the developing market for building materials, including the use of high-performance concrete and innovative solutions for reinforcing concrete structural elements.

**Figure 1:**
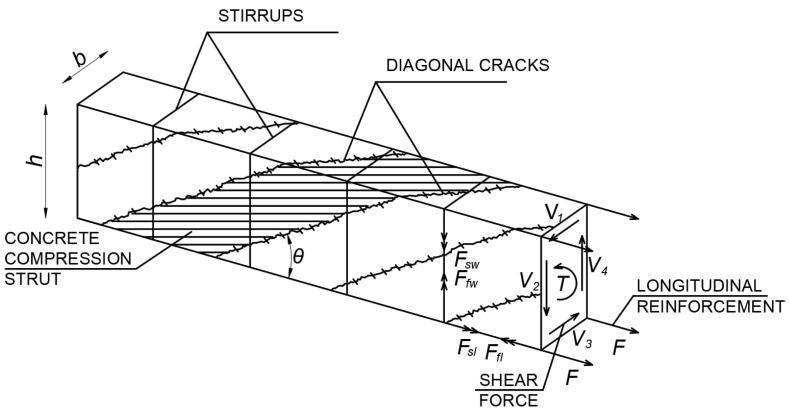
Spatial truss model in torsion calculations.

In order to understand the influence of fibers on torsional load-bearing capacity, one of the most popular calculation models used to calculate reinforced concrete elements under torsion should be presented first (Figure 1). This model is a spatial truss, which, in its assumptions, resembles a truss used in shear calculations. The load-bearing capacity will therefore consist of individual components of the truss model: force *F_sl_* in longitudinal bars placed at the corners of the cross-section, force *F_sw_* in closed-tension stirrups, and additionally, the load-bearing capacity being influenced by a diagonally compressed concrete strut inclined at an angle *θ*. However, when discussing cross-sections made of fiber-reinforced concrete, the truss model described above should be modified. Figure 1 illustrates only those fibers that will be responsible for torsional load-bearing capacity, i.e., fibers that will bridge diagonal cracks. The diagonal arrangement of the fibers will result in a horizontal force *F_fl_* and a vertical force *F_fw_*, as additional factors increasing the torsional resistance. At the same time, according to the results of many studies, after the cracking of the torsion element, the concrete contained inside the cross-section near the center of gravity does not reach its full strength and is not subject to cracking. Hence, similarly to the case of reinforced concrete elements, its contribution is omitted in the calculations, and the cross-section is considered to be a thin-walled box section.

**Figure 2:**
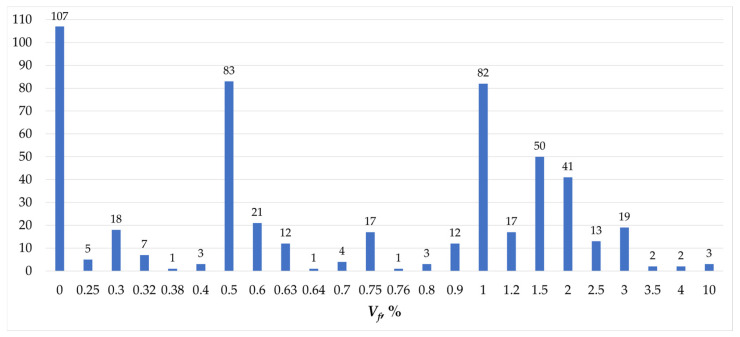
Number of beam elements in which the appropriate amount of fibers is used [1,2,3,4,5,6,7,8,9,10,11,12,13,14,15,16,17,18,19,20,21,22,23,24,25,26,27,28,29,30,31,32,33,34,35,36,37,38,39,40,41,42,43,44,45,46,47,48].

The aim of this review article is to analyze previous research on the torsion of structural elements made of concrete reinforced with dispersed reinforcement. The analysis used various journal databases, including Elsevier, MDPI, BazTech, DOAJ, Ebsco, Infona, ProQuest Central, Springer Link, Web of Science, Wiley Online Library, Hindawi, and Sciendo. A total of approximately 270 articles published between 1979 and 2024 and which fell within the researched thematic area were evaluated (Table A1). Ultimately, based on the analyses conducted by the authors of the referenced articles and the results obtained, 48 articles were selected for analysis in this paper. Figure 2 illustrates the number of beam elements used with the corresponding volume of dispersed reinforcement.

The mechanical properties in terms of compressive strength of composites with the addition of steel fibers were determined in each of the articles [1,2,3,4,5,6,7,8,9,10,11,12,13,14,15,16,17,18,19,20,21,22,23,24,25,26,27,28,29,30,31,32,33,34,35,36,37,38,39,40,41,42,43,44,45,46,47,48] (Table A3). The influence of fiber addition on compressive strength was evident in all cases. Unfortunately, not all mechanical properties were assessed during the tests and presented in the aforementioned articles. Tensile strength was determined in articles [1,3,9,10,12,14,15,16,17,18,19,20,21,22,23,24,26,27,28,29,30,31,32,33,34,35,36,38,39,40,43,46], which demonstrated a positive influence of fiber addition on this property. The modulus of elasticity was analyzed by the following authors: [5,6,11,14,15,16,17,18,19,20,32,34,35,36,38,39,40,45,46,47]. In this case, the impact of the addition was less clear, with either a slight increase or no observable influence of the dispersed reinforcement on this property. Some authors also investigated residual strength [5], where a significant effect of the addition of fibers, along with their percentage content in concrete, was observed on the improvement of the fiber-reinforced concrete class. A few researchers reported the properties of applied longitudinal [1,2,3,5,6,8,9,11,13,19,20,21,22,27,28,32,33,34,36,38,41,43,44,46,47], transverse [1,2,3,5,6,8,19,20,21,22,27,32,33,34,36,38,43,44,46,47], or dispersed [1,3,4,5,6,8,9,10,11,12,16,20,21,25,26,28,29,30,31,32,33,36,38,40,42,43,44,46,48] reinforcement (Table A3). Unfortunately, the absence of basic properties in all analyzed articles means that in several cases it was difficult to draw clear conclusions.

Analysis of the torsion phenomenon was conducted for rectangular elements with an aspect ratio of 1 to 2.5 (Table A2), featuring a longitudinal reinforcement ratio of up to 2.2%, transverse reinforcement of up to 1.7%, and dispersed reinforcement of up to 1.5% (by volume), as well as a composite strength of up to 70 MPa. These criteria were met by 33 articles, which were further analyzed in this article. The authors of the reviewed articles determined the cracking stress (corresponding to the cracking moment) [1,3,4,5,6,7,8,9,11,12,13,14,16,17,18,20,21,22,23,24,27,29,32,33], the ultimate stress (corresponding to the ultimate moment) [1,2,3,4,5,6,7,8,9,10,11,12,13,14,15,16,17,18,19,20,21,22,23,24,25,26,27,28,29,30,31,32,33], the angle of rotation at the cracking moment [1,5,6,7,8,9,11,12,16,17,18,20,22,23,26], the angle of rotation at the maximum moment [1,4,5,6,7,8,9,10,11,12,14,16,17,18,19,20,21,22,23,26,32], the compressive strength [1,2,3,4,5,6,7,8,9,10,11,12,13,14,15,16,17,18,19,20,21,22,23,24,25,26,27,28,29,30,31,32,33], the longitudinal reinforcement ratio [1,2,3,4,5,6,7,8,9,11,12,13,16,17,18,19,20,21,22,23,24,27,28,32,33], and the transverse reinforcement ratio [1,2,3,4,5,7,8,9,12,16,17,18,19,20,21,22,23,24,27,32,33] (Table A4). Additionally, they examined the *h/b* ratio [1,2,3,4,5,6,7,8,9,10,11,12,13,14,15,16,17,18,19,20,21,22,23,24,25,26,27,28,29,30,31,32,33], the slenderness of the element [2,3,4,5,6,7,8,9,11,12,13,15,16,17,19,20,22,27,28,32], and fiber slenderness *l/d* [1,3,4,5,6,8,11,12,13,15,18,19,20,21,22,23,24,25,26,27,28,29,30,31,32,33]. Unfortunately, many articles do not provide information on how the individual values were determined. The lack of this information may affect the results obtained and the conclusions drawn, especially when comparing results obtained by different authors.

All the analyzed articles emphasize the importance of modern building materials in this dynamically evolving field.

This review article attempts to examine and synthesize the latest achievements concerning the behavior of fiber-reinforced concrete beams subjected to torsion. It also comprehensively describes the factors influencing the torsional load-bearing capacity based on previous research, i.e., the compressive strength of concrete *f_c_*, the fiber slenderness *l/d*, the aspect ratio *h/b*, and the slenderness of the tested elements. By using relative torsional stresses (*t_u_/t_u_^o^*), the influence of the fibers themselves on the factors determining this load-bearing capacity for the results of the current research is also presented.

Based on the literature reviewed, it is evident that further research is necessary to gain a more comprehensive understanding of the influence of various factors on the load-bearing capacity of structures under torsional loads, and to develop more advanced theories and models that can be applied in engineering practice. Consequently, this review seeks not only to define the current state of knowledge in this field but also to indicate directions for future research that will contribute to the development of more effective and durable building solutions.

## 2. Results from the Conducted Literature Review

**Figure 3:**
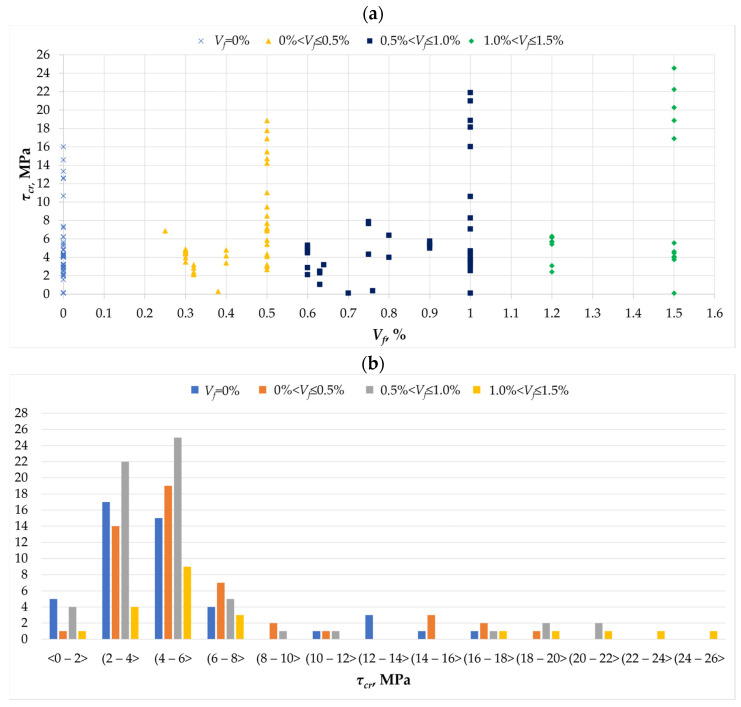
The *τ_cr_* parameter in the analyzed database [1,3,4,5,6,7,8,9,11,12,13,14,16,17,18,20,21,22,23,24,27,29,32,33]: (**a**) the relationship between the cracking stress *τ_cr_* and the amount of dispersed reinforcement *V_f_*; (**b**) histogram of the crack stress *τ_cr_* as a function of the amount of dispersed reinforcement *V_f_*.

Based on the literature review conducted, it is possible to identify the fundamental properties specified by the majority of authors [1,2,3,4,5,6,7,8,9,10,11,12,13,14,15,16,17,18,19,20,21,22,23,24,25,26,27,28,29,30,31,32,33]. First, the description of the results began with the stresses at which a reinforced concrete torsion element cracks (Figure 3a). In the case of reinforced concrete elements without the addition of dispersed reinforcement, the values of cracking stresses reach up to 16 MPa, while the addition of fibers in amounts of up to 0.5% increases this value to over 18 MPa. An amount of fibers in concrete at the level of 1.0% increases the stresses to 22 MPa, while an amount of 1.5% increases the value of cracking stresses to over 24 MPa. However, based on Figure 3b, it can be seen that most researchers achieved values of cracking stresses in the range of 2 to 6 MPa. In this range, the number of conducted tests in which fibers were used in amounts of 0.5% to 1% was the largest. This indicates that the addition of fibers in this amount gained the greatest interest from researchers, which can be explained by the effectiveness of their use in this amount.

Based on the literature review conducted, it can be concluded that the addition of fibers increases the value of the cracking moment compared to the element without dispersed reinforcement. This value increases with the increase in fiber content in the element [1,2,3,4,5,6,7,8,9,10,11,12,13,14,15,16,17,18,19,20,21,22,23,24,25,26,27,28,29,30,31,32,33]. The literature review conducted shows, among other things, that non-dispersed reinforcement increases the cracking moment to a greater extent compared to traditional reinforcement, and that fibers have a greater effect on the cracking moment than on the maximum torsional strength [24].

**Figure 4:**
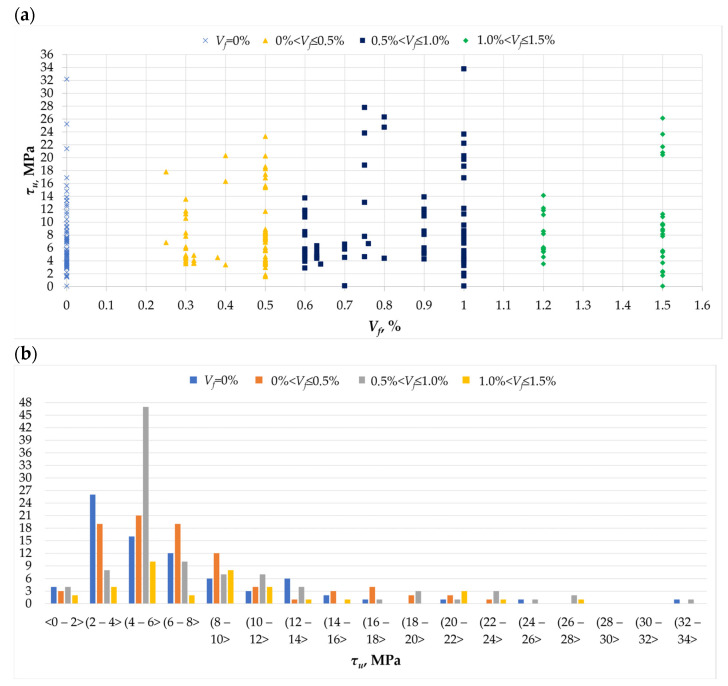
The *τ_u_* parameter in the analyzed database [1,2,3,4,5,6,7,8,9,10,11,12,13,14,15,16,17,18,19,20,21,22,23,24,25,26,27,28,29,30,31,32,33]: (**a**) the relationship between the ultimate stress *τ_u_* and the amount of dispersed reinforcement *V_f_*; (**b**) histogram of the ultimate stress *τ_u_* as a function of the amount of dispersed reinforcement *V_f_*.

Another important feature of torsionally stiffened elements, determined by researchers [1,2,3,4,5,6,7,8,9,10,11,12,13,14,15,16,17,18,19,20,21,22,23,24,25,26,27,28,29,30,31,32,33], is their load-bearing capacity, understood as torsional strength (*τ_u_*). The results of the literature review conducted in the field of the change in the value of torsional strength as a function of the amount of dispersed reinforcement used are presented in Figure 4a. It shows that the highest strength of about 34 MPa was obtained with the addition of fibers in an amount of 1.0%. Some of the researchers obtained relatively high values of torsional strength for elements without dispersed reinforcement, reaching even 32 MPa [4]. This is probably due to the significant amount of additional reinforcement in the tested element. However, most of the researchers obtained lower values of torsional strength (Figure 4b). For elements without dispersed reinforcement, the largest number of analyzed elements (26) yielded values in the range from 2 to 4 MPa. A total of 16 elements obtained stress values in the range from 4 to 6 MPa, while 12 elements were in the range from 6 to 8 MPa. Some researchers added fibers in amounts of up to 0.5%, which resulted in stress values mainly in the range from 2 to 10 MPa. However, the largest number of tested elements contained fibers in the range from 0.5 to 1.0% (47 beams); for this group, the stress value obtained was from 4 to 6 MPa. A relatively small number of the tested elements contained dispersed reinforcement in amounts above 1%, which may indicate the lack of feasibility and effectiveness of using a larger number of fibers in the twisted element.

Source materials show that the addition of fibers increases the torsional strength of beam elements [1,2,3,4,5,6,7,8,9,10,11,12,13,14,15,16,17,18,19,20,21,22,23,24,25,26,27,28,29,30,31,32,33]. The amount of dispersed reinforcement used affects the torsional strength values. With an increase in the volume of fiber used, the torsional strength increases. However, not every amount of fibers improved this value. Fibers in the amount of about 0.3% had a negligible effect on the torsional strength value [20,21,23,24].

**Figure 5:**
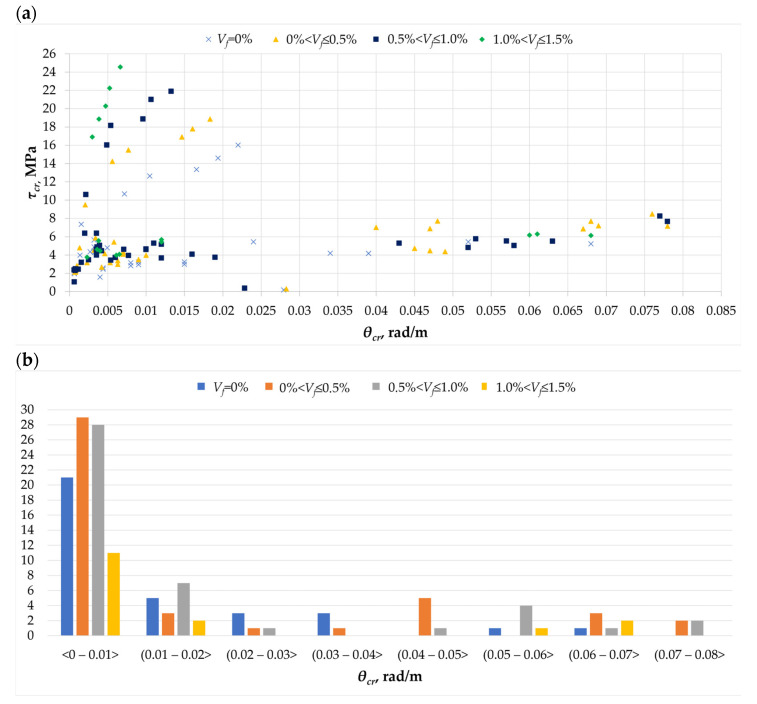
The *θ_cr_* parameter in the analyzed database [1,5,6,7,8,9,11,12,16,17,18,20,22,23,26]: (**a**) the relationship between the crack stress *τ_cr_* and the rotation angle *θ_cr_*; (**b**) histogram of the angle of rotation corresponding to the cracking stress *θ_cr_* for different amounts of dispersed reinforcement in the twisted element.

The torsion phenomenon is always accompanied by an angle of rotation. This can be measured and defined in various ways. In this article, it is assumed to be defined in rad/m. Based on the bibliographic query [1,2,3,4,5,6,7,8,9,10,11,12,13,14,15,16,17,18,19,20,21,22,23,24,25,26,27,28,29,30,31,32,33], it was determined that the angle of rotation changes depending on the stresses causing the cracking of the twisted element (Figure 5a). The highest value of the angle of rotation (*θ_cr_*), about 0.08 rad/m, was obtained for an element containing fibers in an amount of up to 1%. This is a relatively high value compared to most of the presented results. Most researchers obtained a cracking angle of rotation no greater than 0.01 rad/m (Figure 5b). If we consider the results of strength at cracking of the twisted element, in this range, it can be seen that the addition of fibers causes a decrease in the angle of rotation [7]. This is undoubtedly related to the increase in the fracture energy of the twisted element [27].

**Figure 6:**
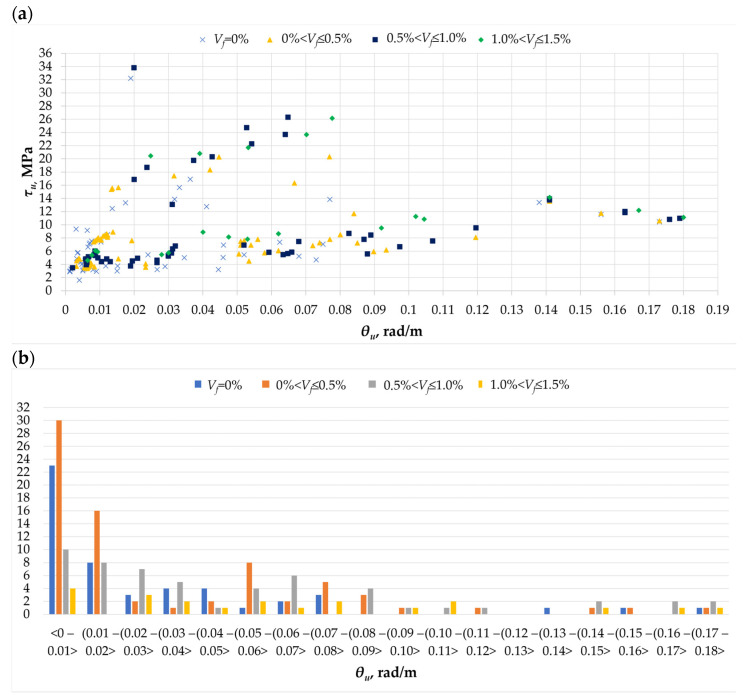
The *θ_u_* parameter in the analyzed database [1,4,5,6,7,8,9,10,11,12,14,16,17,18,19,20,21,22,23,26,32]: (**a**) the relationship between the ultimate stress *τ_cr_* and the rotation angle *θ_u_*; (**b**) histogram of the angle of rotation corresponding to the ultimate stress *θ_u_* for different amounts of dispersed reinforcement in the twisted element.

The rotation angle was also analyzed for the ultimate stresses of the torsion element (Figure 6a). The highest value of the rotation angle (*θ_u_* = 0.18 rad/m) was obtained for the ultimate stresses of about 11 MPa, while the highest stress value (*τ_u_* = 34 MPa) was recorded for a rotation angle of 0.02 rad/m. This discrepancy in results indicates that the addition of fibers can cause an increase in the value of the rotation angle, which is visible in the greater ductility of the material [10,12,25,32], or that the addition of fibers reduces the rotation angle, thus increasing the torsional stiffness and strength of the element [13,21]. At the same time, it can be assumed that the rotation angle can be approximately the same for elements with and without dispersed reinforcement [31]. These discrepancies in results and conclusions drawn can be used to carry out verification tests. Additionally, when analyzing Figure 6b, it can be seen that the largest number of results for the angle of rotation corresponding to the ultimate moment was obtained for the value of 0.01 rad/m, which may indicate that some of the individual results defining significant angles of rotation are burdened with statistical error.

**Figure 7:**
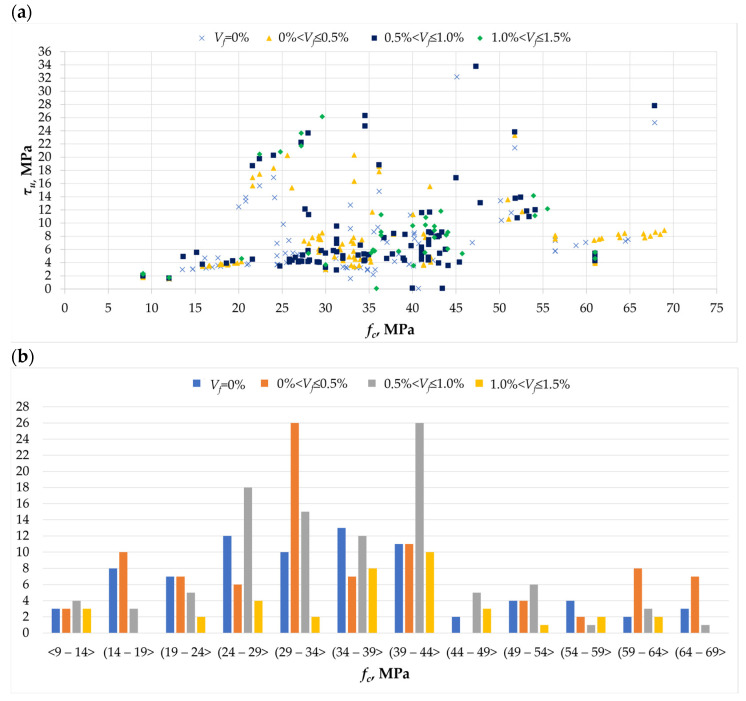
The *f_c_* parameter in the analyzed database [1,2,3,4,5,6,7,8,9,10,11,12,13,14,15,16,17,18,19,20,21,22,23,24,25,26,27,28,29,30,31,32,33]: (**a**) the relationship between the ultimate stress *τ_u_* and the compressive strength *f_c_*; (**b**) histogram of compressive strength *f_c_* for different amounts of dispersed reinforcement.

Compressive strength is a key parameter describing the properties of concrete. Its influence on other features of the cement composite is crucial. The addition of fibers to concrete means that the relationship between compressive strength and, among others, the strength of the torsional element is not clearly defined. The above relationship is presented in Figure 7a, illustrating the influence of the amount of applied dispersed reinforcement, obtained on the basis of works [1,2,3,4,5,6,7,8,9,10,11,12,13,14,15,16,17,18,19,20,21,22,23,24,25,26,27,28,29,30,31,32,33]. It can be seen that compressive strength increases with the amount of applied dispersed reinforcement [3,10,25,29], and at the same time, the torsional strength of the element increases [12,32,33]. As can be seen from Figure 7b, most of the test elements were made of composites with strength in the range of 25 to 45 MPa. The largest number of test elements were made with the addition of steel fibers in amounts from 0 to 0.5% and from 0.5 to 1.0%, obtaining concretes with compressive strengths from 29 to 34 and from 39 to 44 MPa, respectively. Concrete with compressive strength values in these ranges are currently the most popular in residential construction.

**Figure 8:**
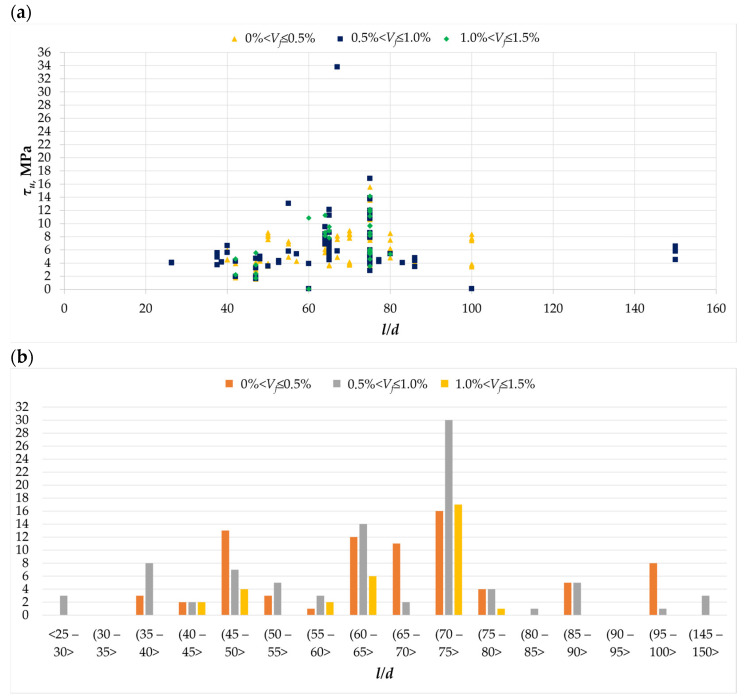
The *l/d* parameter in the analyzed database [1,3,4,5,6,8,11,12,13,15,18,19,20,21,22,23,24,25,26,27,28,29,30,31,32,33]: (**a**) the relationship between the ultimate stress *τ_u_* and the fiber slenderness *l/d*; (**b**) histogram of fiber slenderness *l/d* for different amounts of dispersed reinforcement.

Another important feature influencing the load-bearing capacity of twisted elements is the slenderness of the fibers used. As can be seen from the literature review, the efficiency of fibers depends, among other things, on their dimensions. The slenderness of fibers, i.e., the ratio of length to diameter, is the basic parameter describing them. Figure 8a shows the effect of the slenderness of fibers on the torsional strength of the element. It can be seen that the most effective fibers are those with an *l/d* ratio in the range of 60 to 80. It follows from this that regardless of the number of fibers used, the highest values of torsional load-bearing capacity were achieved in this range. Also, from Figure 8b, it can be concluded that the authors of publications [23,27,28,29] most often used fibers with a slenderness of 60 to 75, which probably resulted from their knowledge of the effect of the slenderness of fibers on the properties of the fresh mixture and the hardened composite [49].

**Figure 9:**
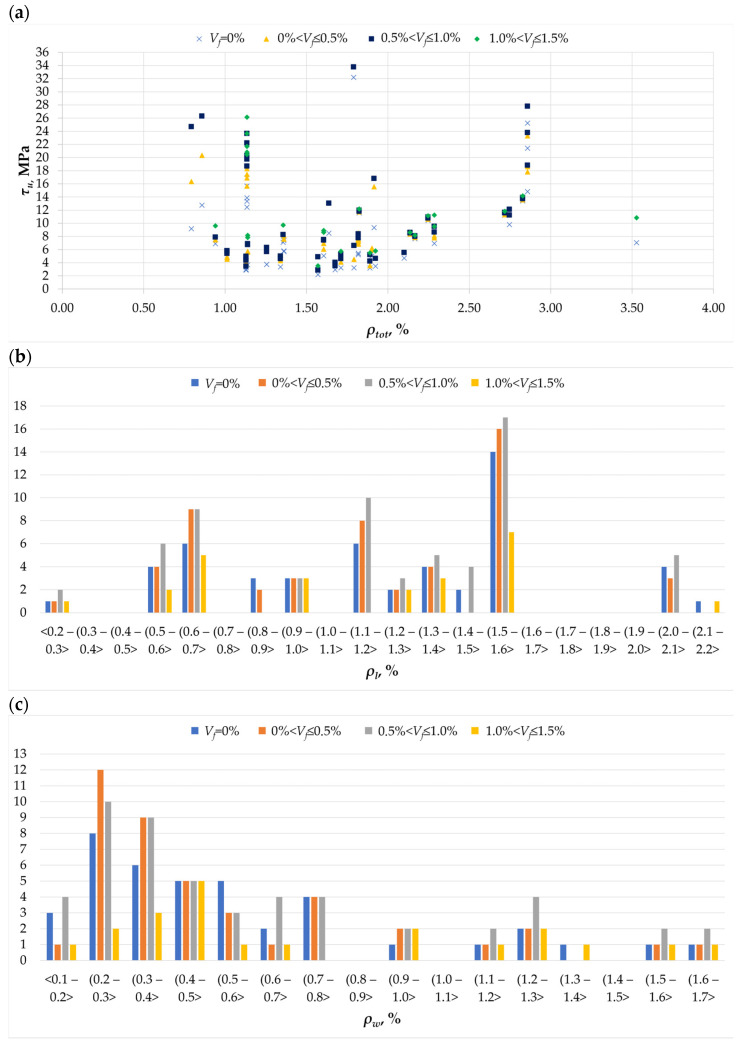
The parameters *ρ_tot_*, *ρ_l_,* and *ρ_w_* in the analyzed database: (**a**) the relationship between the ultimate stress *τ_u_* and total reinforcement ratio *ρ_tot_* [1,2,3,4,5,6,7,8,9,11,12,13,16,17,18,19,20,21,22,23,24,27,28,32,33]: (**b**) histogram of longitudinal reinforcement *ρ_l_* for different amounts of dispersed reinforcement in the twisted element [1,2,3,4,5,6,7,8,9,11,12,13,16,17,18,19,20,21,22,23,24,27,28,32,33]; (**c**) histogram of transverse reinforcement *ρ_w_* for different amounts of dispersed reinforcement in the twisted element [1,2,3,4,5,7,8,9,12,16,17,18,19,20,21,22,23,24,27,32,33].

Conventional reinforcement in torsion elements is the main factor determining their load-bearing capacity. In practically every research work, the influence of the amount of longitudinal and transverse reinforcement on torsional load-bearing capacity was analyzed [50,51]. The situation is similar in the case of beams in which fibers provide additional reinforcement. The histograms (Figure 9b,c) show that the most common longitudinal reinforcement ratio in beams ranged from 1.5% to 1.6%, while the most common transverse reinforcement ratios ranged from 0.2 to 0.4%. It is assumed that the load-bearing capacity of the elements under torsion can be achieved by joint plasticization of the longitudinal and transverse reinforcement—most often in the case of low *ρ_l_* and *ρ_w_* values, by plasticization of the transverse reinforcement—in the case of small *ρ_w_* and large *ρ_l_*, by plasticization of the longitudinal reinforcement where the ratio of reinforcement ratios is inverse, and for large *ρ_l_* and *ρ_w_* values, failure occurs through crushing of the concrete before the yield strength of the longitudinal and transverse reinforcement has been reached [52].

Due to the very complex effects of longitudinal and transverse reinforcement in torsion elements, it was decided to compare torsional stresses to the total longitudinal reinforcement *ρ_l_* and transverse reinforcement *ρ_w_* in the form of total reinforcement ratio *ρ_tot_* in the analysis. A clear trend can be seen in the *τ_u_* to *ρ_tot_* graph (Figure 9a), where the increase in maximum stresses is accompanied by an increase in the reinforcement ratio. It should be noted that the increasing trend is contradicted by the research results from two articles [7,17], where the load-bearing capacity with a small reinforcement ratio reached a value similar to the case of a threefold higher reinforcement ratio in beams, according to other authors. However, it was decided not to reject the research results, thus showing possible variability despite the general trend in the graph. The influence of fiber content on cooperation with conventional reinforcement in transferring torsional stresses is presented in the next section.

**Figure 10:**
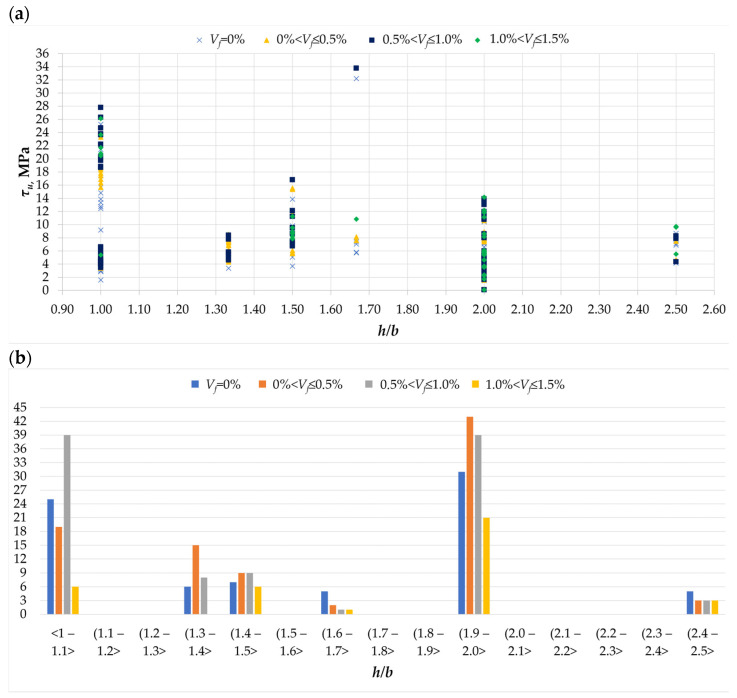
The *h/b* parameter in the analyzed database [1,2,3,4,5,6,7,8,9,10,11,12,13,14,15,16,17,18,19,20,21,22,23,24,25,26,27,28,29,30,31,32,33]: (**a**) the relationship between the ultimate stress *τ_u_* and aspect ratio *h/b*; (**b**) histogram of aspect ratio *h/b* for different amounts of dispersed reinforcement in the twisted element.

The issue of the influence of cross-section dimensions on the torsional resistance of concrete elements was analyzed in the second half of the 20th century. Researchers agree that the aspect ratio of the cross-section has a large influence on the torsional resistance as well as on the change in the angle at which this resistance is achieved [53]. It is stated that square cross-section elements (*h/b* = 1) achieve greater torsional resistance than their counterparts with a cross-section proportion *h/b* > 1. Researchers also agree that torsional resistance decreases with the increase in the height of the cross-section, although no proportionally linear relationship between these two features has been found for *h/b* in the range <1:2>. At the same time, it is noted that with *h/b* > 2, the torsional resistance decreases linearly with the increase in height [52]. Figure 10a shows the dependence of torsional stresses on the cross-section proportions for beams with different fiber content and without fibers. According to the histogram, the most frequently tested elements are beams with a rectangular cross-section *h/b* = 2.0, followed by square cross-sections. Rectangular cross-sections with *h/b* < 2.0 were also noted, but in a relatively smaller number (see Figure 10b). Assuming that the most frequently tested elements are those with cross-sections *h/d* = 2 and *h/d* = 1, a clear trend of decreasing torsional stresses can be observed, and the difference in the achieved stresses is almost twofold: for beams with *h/d* = 1, the maximum stresses in the described beams were 28 MPa, and for beams with a cross-section *h/b* = 2, they were only 14 MPa. At the same time, it should be noted that there is no clear trend in the influence of steel fibers on the analyzed phenomenon, which suggests similar conclusions to those reached in the analysis of the influence of fibers on slenderness.

**Figure 11:**
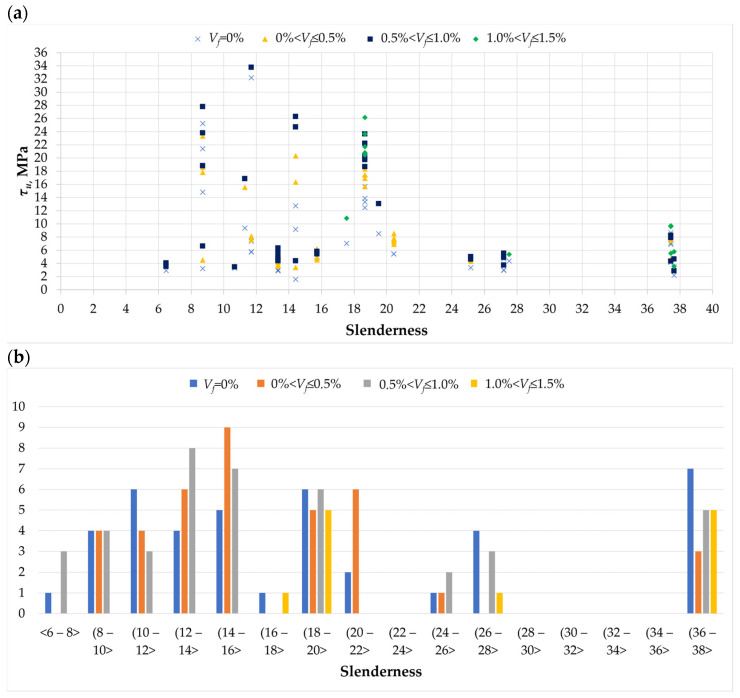
Slenderness in the analyzed database [2,3,4,5,6,7,8,9,11,12,13,15,16,17,19,20,22,27,28,32]: (**a**) the relationship between ultimate stress *τ_u_* and slenderness of twisted elements; (**b**) histogram of slenderness ratios for elements with varying amounts of dispersed reinforcement in the twisted element.

Figure 11a presents the relations between torsional stresses and torsional slenderness. Torsional slenderness is assumed to be the ratio of the support spacing to the radius of gyration of the cross-section under torsional stress. The issue of the influence of torsional slenderness is discussed in the literature, although it is not analyzed as often as, for example, the influence of cross-section dimensions [1]. In general, a tendency is observed in which stockier beams achieve higher torsional load-bearing capacities; the influence of slenderness disappears over time, and for beams with high but variable slenderness, it is essentially unnoticeable. The phenomenon described above is also visible in Figure 11a, but only with a significant increase in slenderness equal to 37–38, where stresses were achieved in the range from 2 to 10 MPa. More stocky beams with slenderness from 8 to 19 showed a similar level of torsional stresses, reaching values of up to 34 MPa, with slenderness of 11.7. Additionally, the scatter of results for stocky beams was noticeably larger than for slender beams. The most commonly adopted slenderness values in the tests were in the range of 12–16 and 36–38. Regarding dispersed reinforcement, it was found that the slenderness effect in beams with fibers is just as noticeable as that in beams without fibers, without the amount of fiber content having a significant influence. Hence, it can be assumed that dispersed reinforcement does not have a major impact on the potential reduction or intensification of the influence of beam slenderness on torsional stresses.

## 3. Analysis of Research Results from Conducted Literature Review

The previous section described the general dependencies of torsional stresses both at the moment of cracking and at the moment of reaching torsional capacity, along with different factors that determine this capacity. However, it should be noted that the described torsional capacity depends on the factors described earlier (i.e., cross-section geometry, reinforcement ratio, matrix compressive strength, or slenderness), and determining the efficiency of fibers in transferring torsional stresses by comparing only the load-bearing capacity values may be problematic. Therefore, it was decided that the fiber effect itself (the effect of fiber reinforcement) would be interpreted by the expression *τ/τ*^0^, in which the torsional stresses of reinforced concrete beams with fibers τ are divided by the torsional stresses of reference reinforced concrete beams without fibers *τ*^0^, with the same degree of reinforcement, cross-section geometry, slenderness, and matrix strength as the beams with fibers. This will allow the factors described above to be eliminated by focusing only on the dispersed reinforcement itself.

**Figure 12:**
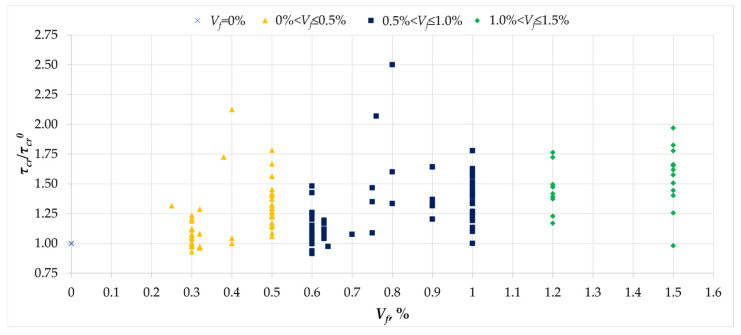
The relationship between the normalized crack stress *τ_cr_/τ_cr_*^0^ and the amount of dispersed reinforcement *V_f_* [1,3,4,5,6,7,8,9,11,12,13,14,16,17,18,20,21,22,23,24,27,29,32,33].

Figure 12 shows the relationship of *τ_cr_/τ_cr_*^0^ to the fiber content. The influence of fibers on the cracking stress is clearly visible, and their quantity alone influences the level of these stresses. It can be assumed that 0.5% fiber content increases the cracking moment by approx. 25%, 1.0% by approx. 40%, and 1.5% by approx. 60%. It should be noted, however, that this is a general upward trend, which did not always occur in the studies. According to the graph, some researchers noted similar or lower values of cracking stress in elements with fibers compared to those without [5,6,11,20,29]. Additionally, it cannot be clearly stated whether the addition of fibers in amounts greater than 1.5% contributes to the achievement of higher cracking moments by beams, due to the small number of results.

**Figure 13:**
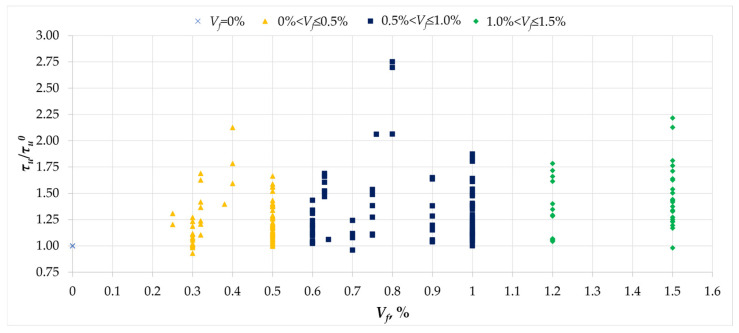
The relationship between the normalized ultimate stress *τ_u_/τ_u_^0^* and the amount of dispersed reinforcement *V_f_* [1,2,3,4,5,6,7,8,9,10,11,12,13,14,15,16,17,18,19,20,21,22,23,24,25,26,27,28,29,30,31,32,33].

Figure 13 illustrates the relationship between the maximum torsional stresses and the maximum stresses in the reference beams, as well as the percentage of fiber content. As in the case of cracking stresses, fibers also have an effect on the torsional load-bearing capacity. This effect is variable and, depending on the test series, it can be marginal, in the order of a few percent, or even more than 2.5 times the load-bearing capacity of the reference beams. It should be noted, however, that no clear trend of increasing load-bearing capacity was observed with increasing fiber content. The reason for this may lie in the use of different types of fibers in the mixtures in terms of shape, the material from which they are made, and their slenderness. It is also worth noting that the high variability of the effect of fiber reinforcement may result from other factors, such as the degree of reinforcement or the compressive strength of the concrete.

**Figure 14:**
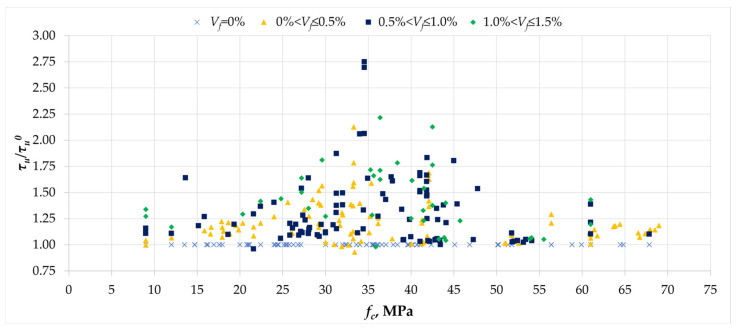
The relationship between the normalized ultimate stress *τ_u_/τ_u_^0^* and the compressive strength *f_c_* [1,2,3,4,5,6,7,8,9,10,11,12,13,14,15,16,17,18,19,20,21,22,23,24,25,26,27,28,29,30,31,32,33].

Figure 14 shows the relationship between the *τ_u_/τ_u_^0^* value and the compressive strength of the concrete used in beam tests. In the literature, there are analyses devoted to the influence of concrete compressive strength on torsional stresses; this influence is obvious, as presented in Figure 7a. However, when analyzing the effect of fiber reinforcement for different concrete strengths, a different trend was observed. The graph shows that regardless of the concrete class, whether in the order of 15 MPa or 65 MPa, fibers provide a minimum reinforcement threshold of about 25% at different levels of dispersed reinforcement. However, the graph also shows that for beams made of concrete with strengths between 25 and 45 MPa, some researchers achieved greater reinforcement effects in the range from 50% to 125%, and their occurrence was independent of the percentage of fiber content. The clear trend of more effective fiber action for concretes in the range of 25–45 MPa may result from the fact that the tensile strength of the matrix is too high in the case of higher concrete classes, which means that the fibers are less effective in transferring tensile stresses, while for concretes of lower strength, the fiber effectiveness may be offset by the generally low strength of the matrix, both in tension and compression, leading to lower values of maximum stresses in the tested elements. Finally, it should be remembered that the scatter of results in the graph may result from other factors mentioned above that affect the torsional load-bearing capacity.

**Figure 15:**
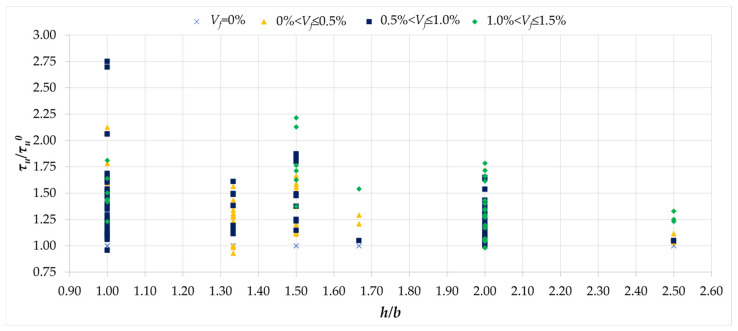
The relationship between the normalized ultimate stress *τ_u_/τ_u_^0^* and aspect ratio *h/b* [1,2,3,4,5,6,7,8,9,10,11,12,13,14,15,16,17,18,19,20,21,22,23,24,25,26,27,28,29,30,31,32,33].

The next figure shows the effect of fiber reinforcement depending on the cross-section dimensions of the tested beams. According to Figure 10a, the cross-sectional aspect ratio has a significant effect on achieving maximum torsional load-bearing capacity, and a similar trend can be observed when analyzing the *τ_u_/τ_u_^0^* values. The influence of fibers for square cross-sections oscillated at a reinforcement level of up to approximately 100%, and in the case of the fiber reinforcement ratio of up to 1.0%, this influence decreased, reaching values of approximately 50%, for *h/b* equal to 2.0. A similar downward trend can be observed for beams reinforced with fibers in the range of 1.0% to 1.5%. Thus, it can be stated that, similarly to the case of torsional load-bearing capacity, the reinforcement effect also depends on the cross-section dimensions; however, when comparing Figure 10a and Figure 15, the decrease in the *τ_u_*/*τ_u_^0^* value is less pronounced.

**Figure 16:**
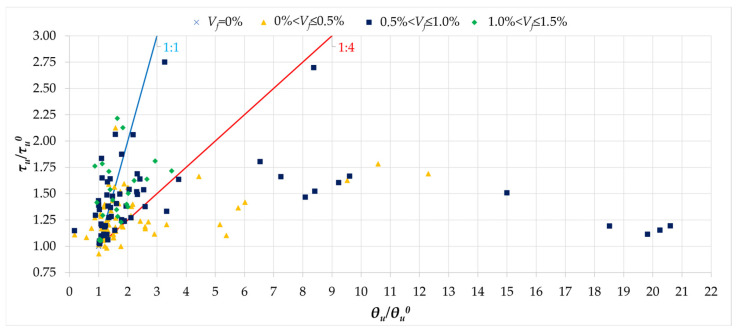
The relationship between the normalized ultimate stress *τ_u_/τ_u_^0^* and normalized rotation angle *θ_u_*/*θ_u_^0^* [1,4,5,6,7,8,9,10,11,12,14,16,17,18,19,20,21,22,23,26,32].

Figure 16 shows the dependence of the *τ_u_/τ_u_^0^* values as a function of the rotation angle ratios for beams with fibers and the rotation angles of reference beams *θ_u_/θ_u_^0^*. According to the analysis presented in the previous section (see Figure 6a), the rotation angles took on different values, which were usually associated with factors such as concrete strength, cross-section dimensions, or reinforcement ratios. In order to better interpret the effect of fibers on the rotation angle, the reinforcement coefficient *τ_u_/τ_u_^0^* and *θ_u_/θ_u_^0^* values were compared, which is intended to help eliminate other factors influencing this angle, and thus the overall assessment of the torsional stiffness of the beams. When analyzing the values of the fiber reinforcement effect, a fairly clear pattern can be observed, where the results can be divided into three groups. The first group, constituting about 7.3% of the analyzed beams, consists of beams in which the addition of fibers resulted in a higher load-bearing capacity compared to the reference beams, while at the same time the torsional stiffness of the beams was greater or at least the same. For this population of results, the influence of fibers proved to be beneficial, improving torsional stiffness. The next group, comprising about 78% of the beams, consists of cases in which a greater increase in the angle of rotation was observed (maximum four times) in relation to the increase in load-bearing capacity, which resulted in a correspondingly lower torsional stiffness. The last group consists of elements for which a significantly greater increase in the angle of rotation was noted with small increases in load-bearing capacity—it is worth emphasizing that the percentage of such beams in the population was small. At the same time, no significant effect of the *V_f_* fiber content on the improvement or deterioration of torsional stiffness was found, and in this case the factor is of secondary importance. It should be noted that in the previous analysis (Figure 5a), it was decided not to compare the effect of fiber reinforcement with the angle of rotation at cracking, due to the negligible influence of fibers on this aspect.

**Figure 17:**
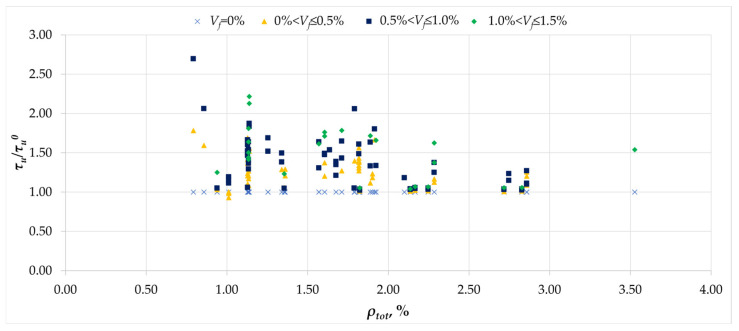
The relationship between the normalized ultimate stress *τ_u_/τ_u_^0^* and total reinforcement ratio *ρ_tot_*.

The relationship between the effect of fiber reinforcement and the reinforcement ratio is illustrated in Figure 17. Given the complex roles of both longitudinal and transverse reinforcement in transferring torsional stresses, the total reinforcement ratio *ρ_tot_* was adopted as the sum of the longitudinal and transverse reinforcement ratios. Figure 17 shows a clear tendency for the reinforcement effect to decrease with increasing *ρ_tot_* value, and this decreasing tendency occurs in each of the ranges of fiber reinforcement ratio *V_f_*. For beams with a reinforcement ratio in the range <1;1.2>, the effects of fiber reinforcement reached values even above 100%, while for *ρ_tot_* equal to 2.75, this effect decreased to approx. 35%. Finally, it can be assumed that an increase in the *ρ_tot_* value by 1% results in a decrease in the fiber reinforcement effect of 50%, and for beams with *ρ_tot_* above 3%, the addition of fibers may become ineffective from the point of view of torsional load capacity.

## 4. Future Research Directions

The analysis of the above-described factors influencing torsion leaves great possibilities for future research directions. The addition of fibers itself has a very random effect on the improvement of torsional load-bearing capacity, and it is possible that other factors contribute in this case to the randomness of the fiber’s influence, i.e., slenderness and aspect ratio. The trends concerning the influence of slenderness or the *h/b* ratio on torsional load-bearing capacity are usually presented for specific tests with appropriate initial assumptions, i.e., the degree of reinforcement and concrete strength. In this case, a number of additional tests should be performed—for example, considering different variants of *h/b* for different degrees of conventional reinforcement, different degrees of slenderness, and different degrees of fiber reinforcement. In a similar way, analysis of torsional slenderness and its influence on conventional reinforcement should be performed. Another aspect in which slenderness may be an important factor is the variable rotation angle. Due to the possible increase in load-bearing capacity, fibers have not always had a positive effect on the improvement of the angle increase, and thus on torsional stiffness. In this case, it would be necessary to perform an analysis of torsional stiffness for different fiber contents as well as for different degrees of slenderness of the elements. It should also be assumed that the interlocking of aggregate in concrete will affect the inclination of the angle of diagonal cracks and thus the subsequent stiffness; hence, performing tests for concretes with different aggregate fractions would be a good complement to the subject of stiffness after the cracking of the element. The above proposals are only the basic directions of research that should be carried out in the future in order to better understand the complex issue of torsion in reinforced concrete elements with fibers.

## 5. Conclusions

The issue of torsion in fiber-reinforced concrete elements is the subject of numerous studies and analyses, in which the authors examine various aspects of this complex phenomenon. Interpreting the previously described results, several conclusions can be drawn:

1. The effect of fibers on the magnitude of torsional cracking stresses can be significant for *V_f_* values greater than 1%.
2. The influence of fiber reinforcement on load-bearing capacity can vary; it may either be minimal or result in nearly double the load-bearing capacity compared to the reference beams. Furthermore, the observed increase in load-bearing capacity does not have a clear correlation with the amount of fibers.
3. The greatest effect of fiber reinforcement on load-bearing capacity was observed for beams made of concrete with strengths ranging from 25 to 45 MPa.
4. The *h/b* cross-section proportions affect the effective performance of fibers. For square cross-sections, the increase in load-bearing capacity due to fibers is greater than that for rectangular cross-sections with an *h/b* ratio of 2.0.
5. Fibers have a small effect on improving torsional stiffness; rather, a reduction in stiffness itself was observed. At the same time, greater ductility was observed for beams that achieved greater rotation angles in relation to the reference beams.
6. The load-bearing capacity gain due to fibers decreased with the increase in the conventional reinforcement ratio, and at *ρ_tot_* above 3% it was marginal.

Despite the considerable amount of research, significant variation in the results has been observed, along with a frequent lack of clear trends in individual analyses, indicating the need for further studies on fiber-reinforced concrete elements subjected to torsion. The above conclusions will allow for the appropriate preparation of a test program for torsion beams regarding element geometry, slenderness, and the amount of conventional reinforcement. Tests on beams made using waste materials are planned; the main material is to be waste fibers (derived from used car tires). Tests using aggregate obtained from ceramic and porcelain waste are also planned, including investigation of the effect of this aggregate together with waste fibers on load-bearing capacity and crack morphology in torsional conditions.

## Data Availability

The raw data supporting the conclusions of this article will be made available by the authors on request.

## Nomenclature

*b*	cross-section width
*d*	fiber diameter
*f_c_*	cylindrical compressive strength
*h*	cross-section height
*h/b*	cross-section aspect ratio
*l*	fiber length
*l/d*	length-to-diameter ratio of the fibers used
*L*	element length
*P*	gauge length
*T_cr_*	cracking moment
*T_u_*	maximum moment
*V_f_*	volumetric percentage of dispersed reinforcement used
*ρ_l_*	longitudinal reinforcement ratio
*ρ_tot_*	degree of conventional reinforcement
*ρ_w_*	transverse reinforcement ratio
*τ_cr_*	shear stresses of the cross-section for the cracking moment
*τ_cr_^0^*	shear stresses of the cross-section for the cracking moment of reference beams
*τ_u_*	shear stresses of the cross-section for the maximum moment
*τ_u_^0^*	shear stresses of the cross-section for the maximum moment of reference beams
*θ*	angle of rotation
*θ_cr_*	angle at the cracking moment
*θ_cr_^0^*	angle at the cracking moment for reference beams
*θ_u_*	angle at the maximum moment
*θ_u_^0^*	angle at the maximum moment for reference beams

## Appendix A

**Table A1:** The parameter ranges and property specifications of the components considered by the authors in their analytical framework.

Characteristics/Properties	Compartment
Aspect ratio *h/b*	from 1 to 2.5
Longitudinal reinforcement ratio, *ρ_l_*	up to 2.2%
Transverse reinforcement, *ρ_w_*	up to 1.7%
Dispersed reinforcement (by volume), *V_f_*	up to 1.5%
Composite strength, *f_c_*	from 9 to 70 MPa
Modulus of elasticity, *E_c_*	from 13 to 50 GPa
Tensile strength, *f_t_*	from 0.1 to 11 MPa

**Table A2:** Geometry of research elements.

Lp.		*b*, mm	*h*, mm	*L*, mm	*P*, mm	*h/b*	Slenderness
[1]	A1-Con	100	150	1500	-	1.50	-
	A1-M13-0.5	100	150	1500	-	1.50	-
	A1-M13-1.0	100	150	1500	-	1.50	-
	A1-M13-1.5	100	150	1500	-	1.50	-
	A1-H35-0.5	100	150	1500	-	1.50	-
	A1-H35-1.0	100	150	1500	-	1.50	-
	A1-H35-1.5	100	150	1500	-	1.50	-
	A2.25-Con	150	225	1500	-	1.50	-
	A2.25-M13-0.5	150	225	1500	-	1.50	-
	A2.25-M13-1.0	150	225	1500	-	1.50	-
	A2.25-M13-1.5	150	225	1500	-	1.50	-
	A2.25-H35-0.5	150	225	1500	-	1.50	-
	A2.25-H35-1.0	150	225	1500	-	1.50	-
	A2.25-H35-1.5	150	225	1500	-	1.50	-
	A4-Con	200	300	1500	-	1.50	-
	A4-M13-0.5	200	300	1500	-	1.50	-
	A4-M13-1.0	200	300	1500	-	1.50	-
	A4-M13-1.5	200	300	1500	-	1.50	-
	A4-H35-0.5	200	300	1500	-	1.50	-
	A4-H35-1.0	200	300	1500	-	1.50	-
	A4-H35-1.5	200	300	1500	-	1.50	-
[2]	B1	200	200	1100	600	1.00	8.00
	B2	200	200	1100	600	1.00	8.00
	B3	200	200	1100	600	1.00	8.00
	B4	200	200	1100	600	1.00	8.00
	B7	200	200	1100	600	1.00	8.00
	B8	200	200	1100	600	1.00	8.00
	B9	200	200	1100	600	1.00	8.00
	B10	200	200	1100	600	1.00	8.00
	B11	200	200	1100	600	1.00	8.00
[3]	B1	250	250	1150	600	1.00	6.40
	B2S	250	250	1150	600	1.00	6.40
	B3H	250	250	1150	600	1.00	6.40
	B4C	250	250	1150	600	1.00	6.40
[4]	Control	150	250	1650	800	1.67	11.69
	1SF	150	250	1650	800	1.67	11.69
[5]	TB1-PC-L18	300	300	2700	1500	1.00	13.33
	TB3-G25-L18	300	300	2700	1500	1.00	13.33
	TB4-G25-L18	300	300	2700	1500	1.00	13.33
	TB5-G50-L18	300	300	2700	1500	1.00	13.33
	TB6-G50-L18	300	300	2700	1500	1.00	13.33
	TB7-D25-L18	300	300	2700	1500	1.00	13.33
	TB8-D25-L18	300	300	2700	1500	1.00	13.33
	TB9-D50-L18	300	300	2700	1500	1.00	13.33
	TB10-D10-L18	300	300	2700	1500	1.00	13.33
	TB11-D50-L18-ST6/150	300	300	2700	1500	1.00	13.33
	TB12-D50-L18-ST6/150	300	300	2700	1500	1.00	13.33
	TB13-PC-L18-ST6/150	300	300	2700	1500	1.00	13.33
	TB14-PC-L18-ST6/150	300	300	2700	1500	1.00	13.33
[6]	TB1-PC	300	300	2700	1500	1.00	13.33
	TB3-SFRC25	300	300	2700	1500	1.00	13.33
	TB4-SFRC25	300	300	2700	1500	1.00	13.33
	TB5-SFRC50	300	300	2700	1500	1.00	13.33
	TB6-SFRC50	300	300	2700	1500	1.00	13.33
[7]	B1	300	300	2500	2100	1.00	18.67
	B2	300	300	2500	2100	1.00	18.67
	B3	300	300	2500	2100	1.00	18.67
	B4	300	300	2500	2100	1.00	18.67
	B5	300	300	2500	2100	1.00	18.67
	B6	300	300	2500	2100	1.00	18.67
	B7	300	300	2500	2100	1.00	18.67
	B8	300	300	2500	2100	1.00	18.67
	B9	300	300	2500	2100	1.00	18.67
	B10	300	300	2500	2100	1.00	18.67
	B11	300	300	2500	2100	1.00	18.67
	B12	300	300	2500	2100	1.00	18.67
	B13	300	300	2500	2100	1.00	18.67
	B14	300	300	2500	2100	1.00	18.67
	B15	300	300	2500	2100	1.00	18.67
	B16	300	300	2500	2100	1.00	18.67
	B17	300	300	2500	2100	1.00	18.67
	B18	300	300	2500	2100	1.00	18.67
	B19	300	300	2500	2100	1.00	18.67
	B20	300	300	2500	2100	1.00	18.67
[8]	T-Reference	200	200	1500	600	1.00	8.00
	T30SF	200	200	1500	600	1.00	8.00
	T60SF	200	200	1500	600	1.00	8.00
[9]	R1	150	250	1300	800	1.67	11.69
	R2	150	250	1300	800	1.67	11.69
	R3	150	250	1300	800	1.67	11.69
	FR2	150	250	1300	800	1.67	11.69
	FR3	150	250	1300	800	1.67	11.69
[10]	A-VC Plain	100	200	2300	-	2.00	-
	A-SFVC 50	100	200	2300	-	2.00	-
	A-SFVC 70	100	200	2300	-	2.00	-
	A-SFVC 100	100	200	2300	-	2.00	-
	A-SCC Plain	100	200	2300	-	2.00	-
	A-SFSCC 50	100	200	2300	-	2.00	-
	A-SFSCC 70	100	200	2300	-	2.00	-
	A-SFSCC 100	100	200	2300	-	2.00	-
	A-RVC Plain	100	200	2300	-	2.00	-
	A-RSFVC 50	100	200	2300	-	2.00	-
	A-RSFVC 70	100	200	2300	-	2.00	-
	A-RSFVC 100	100	200	2300	-	2.00	-
	A-RSCC Plain	100	200	2300	-	2.00	-
	A-RSFSCC 50	100	200	2300	-	2.00	-
	A-RSFSCC 70	100	200	2300	-	2.00	-
	A-RSFSCC 100	100	200	2300	-	2.00	-
	B-VC Plain	100	200	2300	-	2.00	-
	B-SFVC 50	100	200	2300	-	2.00	-
	B-SFVC 70	100	200	2300	-	2.00	-
	B-SFVC 100	100	200	2300	-	2.00	-
	B-SCC Plain	100	200	2300	-	2.00	-
	B-SFSCC 50	100	200	2300	-	2.00	-
	B-SFSCC 70	100	200	2300	-	2.00	-
	B-SFSCC 100	100	200	2300	-	2.00	-
	B-RVC Plain	100	200	2300	-	2.00	-
	B-RSFVC 50	100	200	2300	-	2.00	-
	B-RSFVC 70	100	200	2300	-	2.00	-
	B-RSFVC 100	100	200	2300	-	2.00	-
	B-RSCC Plain	100	200	2300	-	2.00	-
	B-RSFSCC 50	100	200	2300	-	2.00	-
	B-RSFSCC 70	100	200	2300	-	2.00	-
	B-RSFSCC 100	100	200	2300	-	2.00	-
[11]	TB1-PC	300	300	2400	1200	1.00	10.67
	TB2-SFRC25	300	300	2400	1200	1.00	10.67
	TB3-SFRC50	300	300	2400	1200	1.00	10.67
[12]	M0S	200	300	1500	1000	1.50	11.30
	M0.5S	200	300	1500	1000	1.50	11.30
	M1S	200	300	1500	1000	1.50	11.30
[13]	B05-SF0-T	150	200	2000	1600	1.33	25.16
	B06-SF0.5-T	150	200	2000	1600	1.33	25.16
	B07-SF0.75-T	150	200	2000	1600	1.33	25.16
	B08-SF1-T	150	200	2000	1600	1.33	25.16
[14]	NWC	100	150	1100	-	1.50	-
	NWFRC	100	150	1100	-	1.50	-
	CSC	100	150	1100	-	1.50	-
	CSFRC	100	150	1100	-	1.50	-
[15]	MIX-1	140	140	1500	1420	1.00	27.05
	MIX-2	140	140	1500	1420	1.00	27.05
[16]	NWC	150	200	1500	1300	1.33	20.44
	NWFRC-55	150	200	1500	1300	1.33	20.44
	NWFRC-65	150	200	1500	1300	1.33	20.44
	NWFRC-80	150	200	1500	1300	1.33	20.44
	OPSC-0	150	200	1500	1300	1.33	20.44
	OPSFRC-55	150	200	1500	1300	1.33	20.44
	OPSFRC-65	150	200	1500	1300	1.33	20.44
	OPSFRC-80	150	200	1500	1300	1.33	20.44
[17]	Ap	150	150	1185	810	1.00	14.40
	A100	150	150	1185	810	1.00	14.40
	A80	150	150	1185	810	1.00	14.40
	Dp	150	150	1185	810	1.00	14.40
	D100	150	150	1185	810	1.00	14.40
	D80	150	150	1185	810	1.00	14.40
	Ep	150	150	1185	810	1.00	14.40
	E100	150	150	1185	810	1.00	14.40
	E80	150	150	1185	810	1.00	14.40
[18]	T0	150	200	1500	-	1.33	-
	T25	150	200	1500	-	1.33	-
	T50	150	200	1500	-	1.33	-
	T75	150	200	1500	-	1.33	-
	T100	150	200	1500	-	1.33	-
[19]	TR-00	150	250	2200	1200	1.67	17.54
	TS-15	150	250	2200	1200	1.67	17.54
[20]	L08F00V0	150	200	1900	1000	1.33	15.73
	L08F40V3	150	200	1900	1000	1.33	15.73
	L08F40V6	150	200	1900	1000	1.33	15.73
	L08F55V3	150	200	1900	1000	1.33	15.73
	L08F55V6	150	200	1900	1000	1.33	15.73
	L08F67V3	150	200	1900	1000	1.33	15.73
	L08F67V6	150	200	1900	1000	1.33	15.73
	L08F80V3	150	200	1900	1000	1.33	15.73
	L08F80V6	150	200	1900	1000	1.33	15.73
	L12F00V0	150	200	1900	1000	1.33	15.73
	L12F40V3	150	200	1900	1000	1.33	15.73
	L12F80V3	150	200	1900	1000	1.33	15.73
[21]	R50C-P	100	200	2000	-	2.00	-
	R50C-F1	100	200	2000	-	2.00	-
	R50C-F2	100	200	2000	-	2.00	-
	R50C-F3	100	200	2000	-	2.00	-
	R50C-F4	100	200	2000	-	2.00	-
	R50L-P	100	200	2000	-	2.00	-
	R50L-F1	100	200	2000	-	2.00	-
	R50L-F2	100	200	2000	-	2.00	-
	R50L-F3	100	200	2000	-	2.00	-
	R50L-F4	100	200	2000	-	2.00	-
	R50T-P	100	200	2000	-	2.00	-
	R50T-F1	100	200	2000	-	2.00	-
	R50T-F2	100	200	2000	-	2.00	-
	R50T-F3	100	200	2000	-	2.00	-
	R50T-F4	100	200	2000	-	2.00	-
[22]	RP0	100	200	1600	1300	2.00	27.18
	RP1	100	200	1600	1300	2.00	27.18
	RL0	100	200	1600	1300	2.00	27.18
	RL1	100	200	1600	1300	2.00	27.18
	RR0	100	200	1600	1300	2.00	27.18
	RR1	100	200	1600	1300	2.00	27.18
[23]	RL-P	100	200	2200	-	2.00	-
	RL-F1	100	200	2200	-	2.00	-
	RL-F2	100	200	2200	-	2.00	-
	RL-F3	100	200	2200	-	2.00	-
	RL-F4	100	200	2200	-	2.00	-
	RT-P	100	200	2200	-	2.00	-
	RT-F1	100	200	2200	-	2.00	-
	RT-F2	100	200	2200	-	2.00	-
	RT-F3	100	200	2200	-	2.00	-
	RT-F4	100	200	2200	-	2.00	-
[24]	R40C-P	100	200	2000	-	2.00	-
	R40C-F1	100	200	2000	-	2.00	-
	R40C-F2	100	200	2000	-	2.00	-
	R40C-F3	100	200	2000	-	2.00	-
	R40C-F4	100	200	2000	-	2.00	-
	R40L-P	100	200	2000	-	2.00	-
	R40L-F1	100	200	2000	-	2.00	-
	R40L-F2	100	200	2000	-	2.00	-
	R40L-F3	100	200	2000	-	2.00	-
	R40L-F4	100	200	2000	-	2.00	-
	R40T-P	100	200	2000	-	2.00	-
	R40T-F1	100	200	2000	-	2.00	-
	R40T-F2	100	200	2000	-	2.00	-
	R40T-F3	100	200	2000	-	2.00	-
	R40T-F4	100	200	2000	-	2.00	-
[25]	LC1	100	200	700	-	2.00	-
	LC1-0.5-32	100	200	700	-	2.00	-
	LC1-1.0-32	100	200	700	-	2.00	-
	LC1-1.5-32	100	200	700	-	2.00	-
	LC2	100	200	700	-	2.00	-
	LC2-0.5-25	100	200	700	-	2.00	-
	LC2-1.0-25	100	200	700	-	2.00	-
	LC2-1.5-25	100	200	700	-	2.00	-
	LC2-0.5-32	100	200	700	-	2.00	-
	LC2-1.0-32	100	200	700	-	2.00	-
	LC2-1.5-32	100	200	700	-	2.00	-
	NC	100	200	700	-	2.00	-
	NC-0.5-32	100	200	700	-	2.00	-
	NC-1.0-32	100	200	700	-	2.00	-
	NC-1.5-32	100	200	700	-	2.00	-
	HC	100	200	700	-	2.00	-
	HC-0.5-25	100	200	700	-	2.00	-
	HC-1.0-25	100	200	700	-	2.00	-
	HC-1.5-25	100	200	700	-	2.00	-
	HC-0.5-32	100	200	700	-	2.00	-
	HC-1.0-32	100	200	700	-	2.00	-
	HC-1.5-32	100	200	700	-	2.00	-
	HC-0.5-50	100	200	700	-	2.00	-
	HC-1.0-50	100	200	700	-	2.00	-
[26]	P20-P	100	200	2000	-	2.00	-
	P20-F1	100	200	2000	-	2.00	-
	P20-F2	100	200	2000	-	2.00	-
	P20-F3	100	200	2000	-	2.00	-
	P20-F4	100	200	2000	-	2.00	-
	P30-P	100	200	2000	-	2.00	-
	P30-F1	100	200	2000	-	2.00	-
	P30-F2	100	200	2000	-	2.00	-
	P30-F3	100	200	2000	-	2.00	-
	P30-F4	100	200	2000	-	2.00	-
	P40-P	100	200	2000	-	2.00	-
	P40-F1	100	200	2000	-	2.00	-
	P40-F2	100	200	2000	-	2.00	-
	P40-F3	100	200	2000	-	2.00	-
	P40-F4	100	200	2000	-	2.00	-
	P50-P	100	200	2000	-	2.00	-
	P50-F1	100	200	2000	-	2.00	-
	P50-F2	100	200	2000	-	2.00	-
	P50-F3	100	200	2000	-	2.00	-
	P50-F4	100	200	2000	-	2.00	-
[27]	B1	100	200	2000	1800	2.00	37.64
	B2	100	200	2000	1800	2.00	37.64
	B3	100	200	2000	1800	2.00	37.64
	B4	100	200	2000	1800	2.00	37.64
	B5	100	200	2000	1800	2.00	37.64
	B6	100	200	2000	1800	2.00	37.64
[28]	B0.0-0	100	250	2190	1850	2.50	37.04
	B0.5-0	100	250	2190	1850	2.50	37.04
	B1.0-0	100	250	2190	1850	2.50	37.04
	B1.5-0	100	250	2190	1850	2.50	37.04
	B0.0-2a	100	250	2190	1850	2.50	37.04
	B0.0-2b	100	250	2190	1850	2.50	37.04
	B0.5-2	100	250	2190	1850	2.50	37.04
	B1.0-2	100	250	2190	1850	2.50	37.04
	B1.5-2	100	250	2190	1850	2.50	37.04
	B0.0-4a	100	250	2190	1850	2.50	37.04
	B0.0-4b	100	250	2190	1850	2.50	37.04
	B0.5-4	100	250	2190	1850	2.50	37.04
	B1.0-4	100	250	2190	1850	2.50	37.04
	B1.5-4	100	250	2190	1850	2.50	37.04
[29]	P1	152.4	304.8	1778	-	2.00	-
	P2	152.4	304.8	1778	-	2.00	-
	P3	152.4	304.8	1778	-	2.00	-
	P4	152.4	304.8	1778	-	2.00	-
[30]	1	102	102	760	-	1.00	-
	2	102	102	760	-	1.00	-
	3	102	102	760	-	1.00	-
	4	102	102	760	-	1.00	-
	5	102	102	760	-	1.00	-
	6	102	102	760	-	1.00	-
	7	102	102	760	-	1.00	-
	8	102	102	760	-	1.00	-
	9	102	102	760	-	1.00	-
	10	102	102	760	-	1.00	-
	11	102	102	760	-	1.00	-
	12	102	102	760	-	1.00	-
	13	102	102	760	-	1.00	-
	14	102	102	760	-	1.00	-
	15	102	102	760	-	1.00	-
[31]	AP	100	100	1000	-	1.00	-
	BP	100	100	1000	-	1.00	-
	CP	100	100	1000	-	1.00	-
	AF	100	100	1000	-	1.00	-
	BF	100	100	1000	-	1.00	-
	CF	100	100	1000	-	1.00	-
[32]	B1	150	300	2000	1400	2.00	19.52
	B4	150	300	2000	1400	2.00	19.52
[33]	B-NC	100	150	1500	-	1.50	-
	B-SSF-13	100	150	1500	-	1.50	-
	B-HSF-13	100	150	1500	-	1.50	-

*b*—cross-section width, *h*—cross-section height, *L*—element length, *P*—gauge length, *l/d*—length-to-diameter ratio of the fibers used.

**Table A3:** Material data of test elements.

Lp.		*f_c_*, MPa	*f_t_*, MPa	*E_c_*, GPa	*ρ_l_*, %	*f_ys_*, MPa	*ρ_w_*, %	*f_ysw_*, MPa	*l/d*	*V_f_*, %	Type of Fiber	*f_f_*, MPa
[1]	A1-Con	24.408	2.45	-	1.34	455	0.94	455	-	0	-	-
	A1-M13-0.5	33.216	4.88	-	1.34	455	0.94	455	65	0.5	straight	2500
	A1-M13-1.0	41.872	5.75	-	1.34	455	0.94	455	65	1	straight	2500
	A1-M13-1.5	42.504	6.10	-	1.34	455	0.94	455	65	1.5	straight	2500
	A1-H35-0.5	29.216	2.71	-	1.34	455	0.94	455	64	0.5	hooked	900–2200
	A1-H35-1.0	31.288	4.15	-	1.34	455	0.94	455	64	1	hooked	900–2200
	A1-H35-1.5	36.384	4.72	-	1.34	455	0.94	455	64	1.5	hooked	900–2200
	A2.25-Con	24.408	2.45	-	1.23	455	0.38	455	-	0	-	-
	A2.25-M13-0.5	33.216	4.88	-	1.23	455	0.38	455	65	0.5	straight	2500
	A2.25-M13-1.0	41.872	5.75	-	1.23	455	0.38	455	65	1	straight	2500
	A2.25-M13-1.5	42.504	6.10	-	1.23	455	0.38	455	65	1.5	straight	2500
	A2.25-H35-0.5	29.216	2.71	-	1.23	455	0.38	455	64	0.5	hooked	900–2200
	A2.25-H35-1.0	31.288	4.15	-	1.23	455	0.38	455	64	1	hooked	900–2200
	A2.25-H35-1.5	36.384	4.72	-	1.23	455	0.38	455	64	1.5	hooked	900–2200
	A4-Con	24.408	2.45	-	0.92	455	0.22	455	-	0	-	-
	A4-M13-0.5	33.216	4.88	-	0.92	455	0.22	455	65	0.5	straight	2500
	A4-M13-1.0	41.872	5.75	-	0.92	455	0.22	455	65	1	straight	2500
	A4-M13-1.5	42.504	6.10	-	0.92	455	0.22	455	65	1.5	straight	2500
	A4-H35-0.5	29.216	2.71	-	0.92	455	0.22	455	64	0.5	hooked	900–2200
	A4-H35-1.0	31.288	4.15	-	0.92	455	0.22	455	64	1	hooked	900–2200
	A4-H35-1.5	36.384	4.72	-	0.92	455	0.22	455	64	1.5	hooked	900–2200
[2]	B1	36.16	-	-	2.07	520	0.79	460	-	0	-	-
	B2	36.16	-	-	2.07	520	0.79	460	-	0.5	hooked	-
	B3	36.16	-	-	2.07	520	0.79	460	-	0.75	hooked	-
	B4	36.16	-	-	2.07	520	0.79	460	-	0.25	hooked	-
	B7	51.76	-	-	2.07	520	0.79	460	-	0	hooked	-
	B8	51.76	-	-	2.07	520	0.79	460	-	0.5	hooked	-
	B9	51.76	-	-	2.07	520	0.79	460	-	0.75	hooked	-
	B10	67.84	-	-	2.07	520	0.79	460	-	0	-	-
	B11	67.84	-	-	2.07	520	0.79	460	-	0.75	hooked	-
[3]	B1	35.7	4.18	-	1.45	445	0.23	445	-	0	-	
	B2S	44.1	6.27	-	1.45	445	0.23	445	50	1	straight	2850
	B3H	43	8.12	-	1.45	445	0.23	445	60	1	hooked	>1000
	B4C	45.4	8.95	-	1.45	445	0.23	445	83	1	wavy	>700
[4]	Control	45.1	-	-	1.49	-	0.29	-	-	0	-	-
	1SF	47.3	-	-	1.49	-	0.29	-	67	1	-	2100
[5]	TB1-PC-L18	34.80	-	32.3	1.13	512	-	-	-	0	-	-
	TB3-G25-L18	42.08	-	34.2	1.13	512	-	-	86	0.32	hooked	2200
	TB4-G25-L18	42.08	-	34.2	1.13	512	-	-	86	0.32	hooked	2200
	TB5-G50-L18	41.84	-	34.2	1.13	512	-	-	86	0.63	hooked	2200
	TB6-G50-L18	41.84	-	34.2	1.13	512	-	-	86	0.63	hooked	2200
	TB7-D25-L18	41.28	-	34	1.13	512	-	-	65	0.32	hooked	2300
	TB8-D25-L18	41.28	-	34	1.13	512	-	-	65	0.32	hooked	2300
	TB9-D50-L18	41.04	-	34	1.13	512	-	-	65	0.63	hooked	2300
	TB10-D10-L18	41.04	-	34	1.13	512	-	-	65	0.63	hooked	2300
	TB11-D50-L18-ST6/150	41.04	-	34	1.13	512	0.13	523	65	0.63	hooked	2300
	TB12-D50-L18-ST6/150	41.04	-	34	1.13	512	0.13	523	65	0.63	hooked	2300
	TB13-PC-L18-ST6/150	39.60	-	33.6	1.13	512	0.13	523	-	0	-	-
	TB14-PC-L18-ST6/150	39.60	-	33.6	1.13	512	0.13	523	-	0	-	-
[6]	TB1-PC	34.80	-	32.3	1.13	516	-	-	-	0	-	-
	TB3-SFRC25	42.08	-	34.2	1.13	516	-	-	86	0.32	hooked	2200
	TB4-SFRC25	42.08	-	34.2	1.13	516	-	-	86	0.32	hooked	2200
	TB5-SFRC50	41.84	-	34.2	1.13	516	-	-	86	0.63	hooked	2200
	TB6-SFRC50	41.84	-	34.2	1.13	516	-	-	86	0.63	hooked	2200
[7]	B1	24	-	-	0.70	-	0.44	-	-	0	-	-
	B2	22.4	-	-	0.70	-	0.44	-	-	0	-	-
	B3	20.8	-	-	0.70	-	0.44	-	-	0	-	-
	B4	20.8	-	-	0.70	-	0.44	-	-	0	-	-
	B5	20	-	-	0.70	-	0.44	-	-	0	-	-
	B6	25.6	-	-	0.70	-	0.44	-	-	0.5	straight	-
	B7	24	-	-	0.70	-	0.44	-	-	0.5	straight	-
	B8	22.4	-	-	0.70	-	0.44	-	-	0.5	straight	-
	B9	21.6	-	-	0.70	-	0.44	-	-	0.5	straight	-
	B10	21.6	-	-	0.70	-	0.44	-	-	0.5	straight	-
	B11	28	-	-	0.70	-	0.44	-	-	1	straight	-
	B12	27.2	-	-	0.70	-	0.44	-	-	1	straight	-
	B13	24	-	-	0.70	-	0.44	-	-	1	straight	-
	B14	22.4	-	-	0.70	-	0.44	-	-	1	straight	-
	B15	21.6	-	-	0.70	-	0.44	-	-	1	straight	-
	B16	29.6	-	-	0.70	-	0.44	-	-	1.5	straight	-
	B17	27.2	-	-	0.70	-	0.44	-	-	1.5	straight	-
	B18	27.2	-	-	0.70	-	0.44	-	-	1.5	straight	-
	B19	24.8	-	-	0.70	-	0.44	-	-	1.5	straight	-
	B20	22.4	-	-	0.70	-	0.44	-	-	1.5	straight	-
[8]	T-Reference	34	-	-	1.54	420	0.25	420	-	0	-	-
	T30SF	34	-	-	1.54	420	0.25	420	40	0.38	hooked	1100
	T60SF	34	-	-	1.54	420	0.25	420	40	0.76	hooked	1100
[9]	R1	56.4	5.6	-	0.84	500	0.52	200	-	0	-	-
	R2	56.4	5.6	-	0.84	500	0.52	200	-	0	-	-
	R3	56.4	5.6	-	0.84	500	0.52	200	-	0	-	-
	FR2	56.4	5.6	-	0.84	500	0.52	200	67	0.5	hooked	2300
	FR3	56.4	5.6	-	0.84	500	0.52	200	67	0.5	hooked	2300
[10]	A-VC Plain	16.96	2.31	-	-	-	-	-	-	0	-	-
	A-SFVC 50	18.72	2.8	-	-	-	-	-	50	0.5	hooked	275
	A-SFVC 70	19.52	2.75	-	-	-	-	-	70	0.5	hooked	275
	A-SFVC 100	18.08	2.62	-	-	-	-	-	100	0.5	hooked	275
	A-SCC Plain	17.92	2.34	-	-	-	-	-	-	0	-	-
	A-SFSCC 50	19.84	2.71	-	-	-	-	-	50	0.5	hooked	275
	A-SFSCC 70	20.32	2.84	-	-	-	-	-	70	0.5	hooked	275
	A-SFSCC 100	18.8	2.64	-	-	-	-	-	100	0.5	hooked	275
	A-RVC Plain	14.72	2.11	-	-	-	-	-	-	0	-	-
	A-RSFVC 50	16.64	2.46	-	-	-	-	-	50	0.5	hooked	275
	A-RSFVC 70	17.84	2.52	-	-	-	-	-	70	0.5	hooked	275
	A-RSFVC 100	15.84	2.37	-	-	-	-	-	100	0.5	hooked	275
	A-RSCC Plain	16.24	2.15	-	-	-	-	-	-	0	-	-
	A-RSFSCC 50	17.92	2.53	-	-	-	-	-	50	0.5	hooked	275
	A-RSFSCC 70	18.64	2.62	-	-	-	-	-	70	0.5	hooked	275
	A-RSFSCC 100	16.56	2.41	-	-	-	-	-	100	0.5	hooked	275
	B-VC Plain	64.48	4.67	-	-	-	-	-	-	0	-	-
	B-SFVC 50	67.36	5.22	-	-	-	-	-	50	0.5	hooked	275
	B-SFVC 70	68.48	5.36	-	-	-	-	-	70	0.5	hooked	275
	B-SFVC 100	66.72	5.16	-	-	-	-	-	100	0.5	hooked	275
	B-SCC Plain	64.8	4.81	-	-	-	-	-	-	0	-	-
	B-SFSCC 50	67.92	5.52	-	-	-	-	-	50	0.5	hooked	275
	B-SFSCC 70	68.96	5.68	-	-	-	-	-	70	0.5	hooked	275
	B-SFSCC 100	66.56	5.34	-	-	-	-	-	100	0.5	hooked	275
	B-RVC Plain	58.8	4.24	-	-	-	-	-	-	0	-	-
	B-RSFVC 50	61.44	4.92	-	-	-	-	-	50	0.5	hooked	275
	B-RSFVC 70	63.84	5.06	-	-	-	-	-	70	0.5	hooked	275
	B-RSFVC 100	60.88	4.88	-	-	-	-	-	100	0.5	hooked	275
	B-RSCC Plain	59.92	4.56	-	-	-	-	-	-	0	-	-
	B-RSFSCC 50	63.68	5.25	-	-	-	-	-	50	0.5	hooked	275
	B-RSFSCC 70	64.4	5.38	-	-	-	-	-	70	0.5	hooked	275
	B-RSFSCC 100	61.76	4.96	-	-	-	-	-	100	0.5	hooked	275
[11]	TB1-PC	32.32	-	31	1.13	543	-	-	-	0	-	-
	TB2-SFRC25	32.944	-	33	1.13	543	-	-	86	0.32	hooked	>2200
	TB3-SFRC50	24.76	-	28.3	1.13	543	-	-	86	0.64	hooked	>2200
[12]	M0S	36	3.6	-	1.13	-	0.79	-	-	0	-	-
	M0.5S	42	4.6	-	1.13	-	0.79	-	75	0.5	straight	2850
	M1S	45	6	-	1.13	-	0.79	-	75	1	straight	2850
[13]	B05-SF0-T	32	-	-	1.34	550	-	-	-	0	-	-
	B06-SF0.5-T	32	-	-	1.34	550	-	-	48	0.5	hooked	-
	B07-SF0.75-T	32	-	-	1.34	550	-	-	48	0.75	hooked	-
	B08-SF1-T	32	-	-	1.34	550	-	-	48	1	hooked	-
[14]	NWC	25.76	2.69	-	-	-	-	-	-	0	-	-
	NWFRC	35.36	3.206	-	-	-	-	-	-	0.5	-	-
	CSC	24.136	2.35	-	-	-	-	-	-	0	-	-
	CSFRC	26.112	3.18	-	-	-	-	-	-	0.5	-	-
[15]	MIX-1	42.35	4.03	-	-	-	-	-	-	0	-	-
	MIX-2	45.72	5.26	-	-	-	-	-	80	1.5	-	-
[16]	NWC	25.36	3.89	17.23	1.51	-	0.25	-	-	0	-	-
	NWFRC-55	28.08	3.89	19.59	1.51	-	0.25	-	55	0.5	hooked	-
	NWFRC-65	29.2	3.95	21.43	1.51	-	0.25	-	65	0.5	hooked	-
	NWFRC-80	29.44	4.11	20.71	1.51	-	0.25	-	80	0.5	hooked	-
	OPSC-0	26.24	2.83	13.25	1.51	-	0.25	-	-	0	-	-
	OPSFRC-55	27.52	3.63	16.2	1.51	-	0.25	-	55	0.5	hooked	-
	OPSFRC-65	28.48	3.85	14.72	1.51	-	0.25	-	65	0.5	hooked	-
	OPSFRC-80	29.6	3.91	15.48	1.51	-	0.25	-	80	0.5	hooked	-
[17]	Ap	32.84	3.12	24.897	-	-	-	-	-	0	-	-
	A100	32.84	3.12	24.897	0.50	-	0.26	-	-	0	-	-
	A80	32.84	3.12	24.897	0.50	-	0.33	-	-	0	-	-
	Dp	33.29	3.78	25.365	-	-	-	-	-	0.4	hooked	-
	D100	33.29	3.78	25.365	0.50	-	0.26	-	-	0.4	hooked	-
	D80	33.29	3.78	25.365	0.50	-	0.33	-	-	0.4	hooked	-
	Ep	34.54	4.15	26.184	-	-	-	-	-	0.8	hooked	-
	E100	34.54	4.15	26.184	0.50	-	0.26	-	-	0.8	hooked	-
	E80	34.54	4.15	26.184	0.50	-	0.33	-	-	0.8	hooked	-
[18]	T0	27.12	2.59	13.87	1.51	-	0.25	-	-	0	-	-
	T25	31.92	3.73	15.67	1.51	-	0.25	-	64	0.25	hooked	-
	T50	32.96	4.17	16.11	1.51	-	0.25	-	64	0.5	hooked	-
	T75	36.72	4.95	16.29	1.51	-	0.25	-	64	0.75	hooked	-
	T100	37.84	5.47	15.5	1.51	-	0.25	-	64	1	hooked	-
[19]	TR-00	46.86	3.39	-	2.14	505	1.31	466	-	0	-	-
	TS-15	41.51	5.77	-	2.14	505	1.31	466	60	1.5	hooked	-
[20]	L08F00V0	34.8	3.51	31.4	0.67	460	0.34	460	-	0	-	-
	L08F40V3	33.4	3.55	30	0.67	460	0.34	460	40	0.3	hooked	1200
	L08F40V6	31.3	3.35	29.9	0.67	460	0.34	460	40	0.6	hooked	1200
	L08F55V3	31	3.08	27.9	0.67	460	0.34	460	55	0.3	hooked	1200
	L08F55V6	30.9	3.41	29.9	0.67	460	0.34	460	55	0.6	hooked	1200
	L08F67V3	32.7	3.42	31.2	0.67	460	0.34	460	67	0.3	hooked	1200
	L08F67V6	29.5	3.12	28.5	0.67	460	0.34	460	67	0.6	hooked	1200
	L08F80V3	31.9	3.46	27.9	0.67	460	0.34	460	80	0.3	hooked	1200
	L08F80V6	30	3.1	28.7	0.67	460	0.34	460	80	0.6	hooked	1200
	L12F00V0	34.8	3.51	31.4	1.51	460	0.34	460	-	0	-	-
	L12F40V3	31.7	3.58	29.6	1.51	460	0.34	460	40	0.3	hooked	1200
	L12F80V3	31.6	3.56	27.5	1.51	460	0.34	460	80	0.3	hooked	1200
[21]	R50C-P	50.12	2.9	-	1.57	432	1.26	432	-	0	-	-
	R50C-F1	50.95	3.11	-	1.57	432	1.26	432	75	0.3	straight	295
	R50C-F2	51.82	3.336	-	1.57	432	1.26	432	75	0.6	straight	295
	R50C-F3	52.45	3.88	-	1.57	432	1.26	432	75	0.9	straight	295
	R50C-F4	53.9	4.38	-	1.57	432	1.26	432	75	1.2	straight	295
	R50L-P	50.21	2.89	-	1.57	432	0.67	432	-	0	-	-
	R50L-F1	51.05	3.04	-	1.57	432	0.67	432	75	0.3	straight	295
	R50L-F2	52.06	3.51	-	1.57	432	0.67	432	75	0.6	straight	295
	R50L-F3	53.41	3.87	-	1.57	432	0.67	432	75	0.9	straight	295
	R50L-F4	54.07	4.35	-	1.57	432	0.67	432	75	1.2	straight	295
	R50T-P	51.35	2.91	-	0.57	432	1.26	432	-	0	-	-
	R50T-F1	52.6	3.29	-	0.57	432	1.26	432	75	0.3	straight	295
	R50T-F2	53.15	3.71	-	0.57	432	1.26	432	75	0.6	straight	295
	R50T-F3	54.12	4.05	-	0.57	432	1.26	432	75	0.9	straight	295
	R50T-F4	55.51	4.45	-	0.57	432	1.26	432	75	1.2	straight	295
[22]	RP0	13.512	1.48	1.45	-	-	-	-	-	0	-	-
	RP1	15.84	2.05	1.84	-	-	-	-	37.5	1	hooked	-
	RL0	14.696	1.56	1.47	1.57	415	-	-	-	0	-	-
	RL1	13.632	1.79	1.80	1.57	415	-	-	37.5	1	hooked	-
	RR0	16.096	1.6	1.60	1.57	415	0.50	344	-	0	-	-
	RR1	15.168	1.86	2.00	1.57	415	0.50	344	37.5	1	hooked	-
[23]	RL-P	33.6	2.25	-	1.57	-	0.01	-	-	0	-	-
	RL-F1	35.2	2.81	-	1.57	-	0.01	-	75	0.3	straight	-
	RL-F2	37.04	3.27	-	1.57	-	0.01	-	75	0.6	straight	-
	RL-F3	37.7	3.81	-	1.57	-	0.01	-	75	0.9	straight	-
	RL-F4	38.4	4.2	-	1.57	-	0.01	-	75	1.2	straight	-
	RT-P	32.48	2.2	-	0.21	-	1.68	-	-	0	-	-
	RT-F1	33.85	2.46	-	0.21	-	1.68	-	75	0.3	straight	-
	RT-F2	34.44	3.35	-	0.21	-	1.68	-	75	0.6	straight	-
	RT-F3	34.95	3.75	-	0.21	-	1.68	-	75	0.9	straight	-
	RT-F4	35.25	3.95	-	0.21	-	1.68	-	75	1.2	straight	-
[24]	R40C-P	39.74	2.83	-	1.01	-	1.12	-	-	0	-	-
	R40C-F1	40.05	3.46	-	1.01	-	1.12	-	75	0.3	straight	-
	R40C-F2	41.06	3.61	-	1.01	-	1.12	-	75	0.6	straight	-
	R40C-F3	41.98	3.82	-	1.01	-	1.12	-	75	0.9	straight	-
	R40C-F4	43.26	4.1	-	1.01	-	1.12	-	75	1.2	straight	-
	R40L-P	40.16	2.91	-	1.57	-	0.57	-	-	0	-	-
	R40L-F1	41.28	3.14	-	1.57	-	0.57	-	75	0.3	straight	-
	R40L-F2	42.16	3.27	-	1.57	-	0.57	-	75	0.6	straight	-
	R40L-F3	43.37	3.53	-	1.57	-	0.57	-	75	0.9	straight	-
	R40L-F4	44.06	4.07	-	1.57	-	0.57	-	75	1.2	straight	-
	R40T-P	40.21	2.88	-	0.57	-	1.01	-	-	0	-	-
	R40T-F1	41.47	3.46	-	0.57	-	1.01	-	75	0.3	straight	-
	R40T-F2	42.81	3.62	-	0.57	-	1.01	-	75	0.6	straight	-
	R40T-F3	43.06	3.85	-	0.57	-	1.01	-	75	0.9	straight	-
	R40T-F4	43.87	4.09	-	0.57	-	1.01	-	75	1.2	straight	-
[25]	LC1	12	-	-	-	-	-	-	-	0	-	-
	LC1-0.5-32	12	-	-	-	-	-	-	47	0.5	-	650
	LC1-1.0-32	12	-	-	-	-	-	-	47	1	-	650
	LC1-1.5-32	12	-	-	-	-	-	-	47	1.5	-	650
	LC2	9	-	-	-	-	-	-	-	0	-	-
	LC2-0.5-25	9	-	-	-	-	-	-	42	0.5	-	650
	LC2-1.0-25	9	-	-	-	-	-	-	42	1	-	650
	LC2-1.5-25	9	-	-	-	-	-	-	42	1.5	-	650
	LC2-0.5-32	9	-	-	-	-	-	-	47	0.5	-	650
	LC2-1.0-32	9	-	-	-	-	-	-	47	1	-	650
	LC2-1.5-32	9	-	-	-	-	-	-	47	1.5	-	650
	NC	30	-	-	-	-	-	-	-	0	-	-
	NC-0.5-32	30	-	-	-	-	-	-	47	0.5	-	650
	NC-1.0-32	30	-	-	-	-	-	-	47	1	-	650
	NC-1.5-32	30	-	-	-	-	-	-	47	1.5	-	650
	HC	61	-	-	-	-	-	-	-	0	-	-
	HC-0.5-25	61	-	-	-	-	-	-	42	0.5	-	650
	HC-1.0-25	61	-	-	-	-	-	-	42	1	-	650
	HC-1.5-25	61	-	-	-	-	-	-	42	1.5	-	650
	HC-0.5-32	61	-	-	-	-	-	-	47	0.5	-	650
	HC-1.0-32	61	-	-	-	-	-	-	47	1	-	650
	HC-1.5-32	61	-	-	-	-	-	-	47	1.5	-	650
	HC-0.5-50	61	-	-	-	-	-	-	57	0.5	-	650
	HC-1.0-50	61	-	-	-	-	-	-	57	1	-	650
[26]	P20-P	17.6	2.35	-	-	-	-	-	-	0	-	-
	P20-F1	17.928	2.54	-	-	-	-	-	75	0.3	straight	275
	P20-F2	18.608	2.75	-	-	-	-	-	75	0.6	straight	275
	P20-F3	19.32	2.82	-	-	-	-	-	75	0.9	straight	275
	P20-F4	20.328	3	-	-	-	-	-	75	1.2	straight	275
	P30-P	25.488	2.7	-	-	-	-	-	-	0	-	-
	P30-F1	25.928	3.05	-	-	-	-	-	75	0.3	straight	275
	P30-F2	26.528	3.29	-	-	-	-	-	75	0.6	straight	275
	P30-F3	27.368	3.71	-	-	-	-	-	75	0.9	straight	275
	P30-F4	28.008	3.89	-	-	-	-	-	75	1.2	straight	275
	P40-P	31.192	2.95	-	-	-	-	-	-	0	-	-
	P40-F1	33.232	3.39	-	-	-	-	-	75	0.3	straight	275
	P40-F2	33.76	3.52	-	-	-	-	-	75	0.6	straight	275
	P40-F3	34.416	3.81	-	-	-	-	-	75	0.9	straight	275
	P40-F4	35.448	3.99	-	-	-	-	-	75	1.2	straight	275
	P50-P	41.048	3.06	-	-	-	-	-	-	0	-	-
	P50-F1	41.904	3.45	-	-	-	-	-	75	0.3	straight	275
	P50-F2	43.128	3.8	-	-	-	-	-	75	0.6	straight	275
	P50-F3	43.8	3.98	-	-	-	-	-	75	0.9	straight	275
	P50-F4	44.04	4.33	-	-	-	-	-	75	1.2	straight	275
[27]	B1	35.47	4.26	-	1.57	250	-	-	-	0	-	-
	B2	31.26	5.94	-	1.57	250	-	-	75	0.6	hooked	-
	B3	40.11	7.58	-	1.57	250	-	-	75	1.2	hooked	-
	B4	40.12	4.18	-	1.57	250	0.35	250	-	0	-	-
	B5	38.93	5.67	-	1.57	250	0.35	250	75	0.6	hooked	-
	B6	35.64	7.68	-	1.57	250	0.35	250	75	1.2	hooked	-
[28]	B0.0-0	37.93	3.14	-	260	260	260	260	-	0	-	-
	B0.5-0	35.1	4.05	-	260	260	260	260	75	0.5	hooked	260
	B1.0-0	39.08	5.13	-	260	260	260	260	75	1	hooked	260
	B1.5-0	41.42	5.67	-	260	260	260	260	75	1.5	hooked	260
	B0.0-2a	40.72	3.15	-	0.90	-	-	-	-	0	-	-
	B0.0-2b	36.59	3.22	-	0.90	-	-	-	-	0	-	-
	B0.5-2	34.16	3.94	-	0.90	-	-	-	75	0.5	hooked	260
	B1.0-2	42.89	5.52	-	0.90	-	-	-	75	1	hooked	260
	B1.5-2	40.01	6.06	-	0.90	-	-	-	75	1.5	hooked	260
	B0.0-4a	37.07	2.33	-	1.36	-	-	-	-	0	-	-
	B0.0-4b	35.56	3.19	-	1.36	-	-	-	-	0	-	-
	B0.5-4	37.8	4.14	-	1.36	-	-	-	75	0.5	hooked	260
	B1.0-4	39.14	4.73	-	1.36	-	-	-	75	1	hooked	260
	B1.5-4	41.48	5.83	-	1.36	-	-	-	75	1.5	hooked	260
[29]	P1	40.68	3.45	-	-	-	-	-	-	0	-	-
	P2	39.99	5.72	-	-	-	-	-	60	0.7	hooked	930–1380
	P3	43.44	6.34	-	-	-	-	-	100	1	hooked	930–1380
	P4	35.85	5.31	-	-	-	-	-	60	1.5	hooked	930–1380
[30]	1	21.12	2.67	-	-	-	-	-	-	0	-	-
	2	20.96	2.8	-	-	-	-	-	-	0	-	-
	3	24.48	2.6	-	-	-	-	-	-	0	-	-
	4	29.04	3.32	-	-	-	-	-	26	1	straight	300
	5	25.84	3.32	-	-	-	-	-	26	1	straight	300
	6	29.28	3.55	-	-	-	-	-	26	1	straight	300
	7	28	3.7	-	-	-	-	-	39	1	straight	300
	8	27.28	3.78	-	-	-	-	-	39	1	straight	300
	9	28	4.04	-	-	-	-	-	39	1	straight	300
	10	27.28	3.92	-	-	-	-	-	53	1	straight	300
	11	28.16	3.83	-	-	-	-	-	53	1	straight	300
	12	26.88	3.95	-	-	-	-	-	53	1	straight	300
	13	25.84	3.99	-	-	-	-	-	77	1	straight	300
	14	27.12	3.76	-	-	-	-	-	77	1	straight	300
	15	26.16	3.8	-	-	-	-	-	77	1	straight	300
[31]	AP	38.4	3.7	-	-	-	-	-	-	0	-	
	BP	26.8	2.8	-	-	-	-	-	-	0	-	
	CP	17.6	2.2	-	-	-	-	-	-	0	-	
	AF	39.84	4.2	-	-	-	-	-	150	0.7	straight	2000
	BF	28	3.5	-	-	-	-	-	150	0.7	straight	2000
	CF	21.6	2.7	-	-	-	-	-	150	0.7	straight	2000
[32]	B1	40.2	4.2	-	1.24	440.5 465.2	0.39	418.6	-	0	-	-
	B4	47.8	4.2	-	1.24	440.5 465.2	0.39	418.6	55	0.75	wavy	2400
[33]	B-NC	25.128	2.73	-	2.09	462	0.65	437	-	0	-	
	B-SSF-13	27.64	3.24	-	2.09	462	0.65	437	65	1	straight	<2100
	B-HSF-13	28.064	3.31	-	2.09	462	0.65	437	65	1	hooked	<2100

*f_c_*—cylindrical compressive strength, *f_t_*—tensile strength, *E_c_*—modulus of elasticity, *ρ_l_*—longitudinal reinforcement ratio, *f_ys_*—yield strength of longitudinal reinforcement, *ρ_w_*—transverse reinforcement ratio, *f_ysw_*—plastic limit of stirrups, *l/d*—length-to-diameter ratio of the fibers used, *V_f_*—volumetric percentage of dispersed reinforcement used, *f_f_*—tensile strength of fibers.

**Table A4:** Torsion test results of individual elements.

Lp.		*T_cr_*, kNm	*Ø_cr_*, rad/m	*Ø_cr_*, rad	*Ø_cr_/Ø_cr_^0^*	*τ_cr_*, MPa	*τ_cr_/τ_cr_^0^*	*T_u_*, kNm	*Ø_u_*, rad/m	*Ø_u_*, rad	*Ø_u_/Ø_u_^0^*	*τ_u_*, MPa	*τ_u_/τ_u_^0^*	*τ_u_/τ_cr_*
[1]	A1-Con	0.9	0.0044	-	1.00	2.60	1.00	2.4	0.046	-	1.00	6.93	1.00	2.67
	A1-M13-0.5	1.1	0.0023	-	0.52	3.17	1.22	2.7	0.056	-	1.22	7.79	1.13	2.45
	A1-M13-1.0	1.2	0.0025	-	0.57	3.46	1.33	3	0.0826	-	1.80	8.66	1.25	2.50
	A1-M13-1.5	1.3	0.0023	-	0.52	3.75	1.44	3.3	0.092	-	2.00	9.52	1.38	2.54
	A1-H35-0.5	1.5	0.007	-	1.59	4.33	1.67	2.8	0.1195	-	2.60	8.08	1.17	1.87
	A1-H35-1.0	1.6	0.0071	-	1.61	4.62	1.78	3.3	0.1196	-	2.60	9.52	1.38	2.06
	A1-H35-1.5	1.6	0.0036	-	0.82	4.62	1.78	3.9	0.102	-	2.22	11.26	1.63	2.44
	A2.25-Con	3.3	0.008	-	1.00	2.82	1.00	5.9	0.0459	-	1.00	5.05	1.00	1.79
	A2.25-M13-0.5	4.8	0.007	-	0.88	4.10	1.45	8.1	0.054	-	1.18	6.93	1.37	1.69
	A2.25-M13-1.0	5.2	0.0042	-	0.53	4.45	1.58	8.7	0.068	-	1.48	7.44	1.47	1.67
	A2.25-M13-1.5	6.5	0.0038	-	0.48	5.56	1.97	10.4	0.04	-	0.87	8.89	1.76	1.60
	A2.25-H35-0.5	3.5	0.0063	-	0.79	2.99	1.06	7.1	0.062	-	1.35	6.07	1.20	2.03
	A2.25-H35-1.0	4.6	0.0077	-	0.96	3.93	1.39	8.8	0.107	-	2.33	7.52	1.49	1.91
	A2.25-H35-1.5	5.2	0.004	-	0.50	4.45	1.58	10.1	0.062	-	1.35	8.64	1.71	1.94
	A4-Con	6.8	0.0044	-	1.00	2.45	1.00	10.2	0.029	-	1.00	3.68	1.00	1.50
	A4-M13-0.5	8.8	0.0053	-	1.20	3.17	1.29	15.9	0.058	-	2.00	5.74	1.56	1.81
	A4-M13-1.0	10.3	0.006	-	1.36	3.72	1.51	18.7	0.032	-	1.10	6.75	1.83	1.82
	A4-M13-1.5	11.3	0.0065	-	1.48	4.08	1.66	21.7	0.053	-	1.83	7.83	2.13	1.92
	A4-H35-0.5	7.4	0.0042	-	0.95	2.67	1.09	15.5	0.0505	-	1.74	5.59	1.52	2.09
	A4-H35-1.0	9.5	0.0054	-	1.23	3.43	1.40	19.1	0.052	-	1.79	6.89	1.87	2.01
	A4-H35-1.5	11	0.0061	-	1.39	3.97	1.62	22.6	0.0475	-	1.64	8.15	2.22	2.05
[2]	B1	-	-	-	-	-	-	24.68	-	0.0192	1.00	14.82	1.00	-
	B2	-	-	-	-	-	-	30.99	-	0.0253	1.32	18.61	1.26	-
	B3	-	-	-	-	-	-	31.37	-	0.026	1.35	18.83	1.27	-
	B4	-	-	-	-	-	-	29.69	-	0.024	1.25	17.83	1.20	-
	B7	-	-	-	-	-	-	35.66	-	0.026	1.00	21.41	1.00	-
	B8	-	-	-	-	-	-	38.83	-	0.032	1.23	23.31	1.09	-
	B9	-	-	-	-	-	-	39.64	-	0.0333	1.28	23.80	1.11	-
	B10	-	-	-	-	-	-	42.04	-	0.0325	1.00	25.24	1.00	-
	B11	-	-	-	-	-	-	46.29	-	0.039	1.20	27.79	1.10	-
[3]	B1	6.56	-	0.0171	1.00	2.02	1.00	9.5	-	0.0382	1.00	2.92	1.00	1.45
	B2S	8.25	-	0.0206	1.20	2.54	1.26	11.5	-	0.0414	1.08	3.54	1.21	1.39
	B3H	9.35	-	0.0237	1.39	2.87	1.43	12.8	-	0.0393	1.03	3.93	1.35	1.37
	B4C	9.86	-	0.0251	1.47	3.03	1.50	13.2	-	0.0384	1.00	4.06	1.39	1.34
[4]	Control	8.3	-	-	-	6.24	1.00	42.8	0.019	-	1.00	32.20	1.00	5.16
	1SF	9.4	-	-	-	7.07	1.13	44.9	0.02	-	1.05	33.78	1.05	4.78
[5]	TB1-PC-L18	12.4	0.0008	-	1.00	2.21	1.00	16.8	0.0013	-	1.00	2.99	1.00	1.35
	TB3-G25-L18	13.4	0.0009	-	1.13	2.38	1.08	27.32	0.0124	-	9.52	4.86	1.63	2.04
	TB4-G25-L18	12	0.0008	-	1.03	2.13	0.97	22.94	0.0075	-	5.78	4.08	1.37	1.91
	TB5-G50-L18	13.89	0.0008	-	1.01	2.47	1.12	26.94	0.012	-	9.23	4.79	1.60	1.94
	TB6-G50-L18	13.74	0.0012	-	1.50	2.44	1.11	24.63	0.0105	-	8.09	4.38	1.47	1.79
	TB7-D25-L18	11.92	0.0007	-	0.87	2.12	0.96	20.27	0.0067	-	5.15	3.61	1.21	1.70
	TB8-D25-L18	15.96	0.001	-	1.26	2.84	1.29	20.8	0.0032	-	2.42	3.70	1.24	1.30
	TB9-D50-L18	13.23	0.0006	-	0.78	2.35	1.07	27.9	0.0094	-	7.25	4.96	1.66	2.11
	TB10-D10-L18	12.9	0.0008	-	0.96	2.29	1.04	25.3	0.0195	-	15.00	4.50	1.51	1.96
	TB11-D50-L18-ST6/150	13.2	0.0007	-	1.05	1.05	1.17	35.6	0.0312	-	2.33	6.33	1.69	2.70
	TB12-D50-L18-ST6/150	13.5	0.0008	-	1.31	2.40	1.19	32	0.0309	-	2.30	5.69	1.52	2.37
	TB13-PC-L18-ST6/150	11.6	0.0006	-	1.00	2.06	1.00	21.11	0.0117	-	1.00	3.76	1.00	1.82
	TB14-PC-L18-ST6/150	11	0.0006	-	1.00	1.96	1.00	21.07	0.0152	-	1.00	3.75	1.00	1.92
[6]	TB1-PC	12.4	0.0008	-	1.00	2.21	1.00	16.18	0.0013	-	1.00	2.88	1.00	1.30
	TB3-SFRC25	13.4	0.0009	-	1.13	2.38	1.08	27.32	0.0154	-	12.30	4.86	1.69	2.04
	TB4-SFRC25	12	0.0008	-	1.03	2.13	0.97	22.94	0.0075	-	6.02	4.08	1.42	1.91
	TB5-SFRC50	13.89	0.0008	-	1.01	2.47	1.12	26.94	0.012	-	9.60	4.79	1.67	1.94
	TB6-SFRC50	13.74	0.0012	-	1.50	2.44	1.11	24.63	0.0105	-	8.42	4.38	1.52	1.79
[7]	B1	90	0.022	-	1.00	16.01	1.00	95	0.0363	-	1.00	16.90	1.00	1.06
	B2	82	0.0194	-	1.00	14.59	1.00	88	0.0332	-	1.00	15.65	1.00	1.07
	B3	75	0.0166	-	1.00	13.34	1.00	78	0.0318	-	1.00	13.88	1.00	1.04
	B4	71	0.0105	-	1.00	12.63	1.00	75	0.0175	-	1.00	13.34	1.00	1.06
	B5	60	0.0072	-	1.00	10.67	1.00	70	0.0136	-	1.00	12.45	1.00	1.17
	B6	106	0.0183	-	1.21	18.86	1.40	114	0.0447	-	1.69	20.28	1.40	1.08
	B7	100	0.0161	-	1.06	17.79	1.32	103	0.0421	-	1.59	18.32	1.27	1.03
	B8	95	0.0147	-	0.97	16.90	1.26	98	0.0316	-	1.19	17.43	1.21	1.03
	B9	87	0.0077	-	0.51	15.48	1.15	95	0.0199	-	0.75	16.90	1.17	1.09
	B10	80	0.0056	-	0.37	14.23	1.06	88	0.0154	-	0.58	15.65	1.08	1.10
	B11	123	0.0133	-	0.88	21.88	1.63	133	0.0641	-	2.42	23.66	1.64	1.08
	B12	118	0.0107	-	0.70	20.99	1.56	125	0.0543	-	2.05	22.24	1.54	1.06
	B13	106	0.0096	-	0.64	18.86	1.40	114	0.0428	-	1.62	20.28	1.40	1.08
	B14	102	0.0054	-	0.36	18.14	1.35	111	0.0374	-	1.41	19.75	1.37	1.09
	B15	90	0.0049	-	0.32	16.01	1.19	105	0.0237	-	0.90	18.68	1.29	1.17
	B16	138	0.0066	-	0.44	24.55	1.83	147	0.0777	-	2.94	26.15	1.81	1.07
	B17	125	0.0052	-	0.35	22.24	1.65	133	0.0702	-	2.65	23.66	1.64	1.06
	B18	114	0.0047	-	0.31	20.28	1.51	122	0.0532	-	2.01	21.70	1.50	1.07
	B19	106	0.0038	-	0.25	18.86	1.40	117	0.0391	-	1.48	20.81	1.44	1.10
	B20	95	0.003	-	0.20	16.90	1.26	115	0.0248	-	0.94	20.46	1.42	1.21
[8]	T-Reference	0.29	0.0279	-	1.00	0.17	1.00	5.37	0.0445	-	1.00	3.22	1.00	18.52
	T30SF	0.5	0.0283	-	1.01	0.30	1.72	7.5	0.0534	-	1.20	4.50	1.40	15.00
	T60SF	0.6	0.0229	-	0.82	0.36	2.07	11.06	0.0974	-	2.19	6.64	2.06	18.43
[9]	R1	5.7	0.0033	-	1.00	4.29	1.00	9.8	0.0103	-	1.00	7.37	1.00	1.72
	R2	5.8	0.0027	-	1.00	4.36	1.00	7.6	0.0035	-	1.00	5.72	1.00	1.31
	R3	7.5	0.0032	-	1.00	5.64	1.00	7.7	0.0036	-	1.00	5.79	1.00	1.03
	FR2	7.2	0.0058	-	1.89	5.42	1.14	10.1	0.0193	-	3.33	7.60	1.21	1.40
	FR3	7.8	0.0034	-	1.11	5.87	1.23	10.8	0.0122	-	2.10	8.12	1.29	1.38
[10]	A-VC Plain	-	-	-	-	-	-	1.63	0.0055	-	1.00	3.31	1.00	-
	A-SFVC 50	-	-	-	-	-	-	1.8	0.0064	-	1.18	3.66	1.10	-
	A-SFVC 70	-	-	-	-	-	-	1.93	0.0068	-	1.25	3.92	1.18	-
	A-SFVC 100	-	-	-	-	-	-	1.84	0.0063	-	1.15	3.74	1.13	-
	A-SCC Plain	-	-	-	-	-	-	1.69	0.0057	-	1.00	3.44	1.00	-
	A-SFSCC 50	-	-	-	-	-	-	1.93	0.0071	-	1.25	3.92	1.14	-
	A-SFSCC 70	-	-	-	-	-	-	2.04	0.0075	-	1.31	4.15	1.21	-
	A-SFSCC 100	-	-	-	-	-	-	1.87	0.0069	-	1.21	3.80	1.11	-
	A-RVC Plain	-	-	-	-	-	-	1.49	0.005	-	1.00	3.03	1.00	-
	A-RSFVC 50	-	-	-	-	-	-	1.74	0.0055	-	1.10	3.54	1.17	-
	A-RSFVC 70	-	-	-	-	-	-	1.82	0.0061	-	1.23	3.70	1.22	-
	A-RSFVC 100	-	-	-	-	-	-	1.69	0.0057	-	1.15	3.44	1.13	-
	A-RSCC Plain	-	-	-	-	-	-	1.56	0.0051	-	1.00	3.17	1.00	-
	A-RSFSCC 50	-	-	-	-	-	-	1.82	0.0064	-	1.26	3.70	1.17	-
	A-RSFSCC 70	-	-	-	-	-	-	1.89	0.0068	-	1.32	3.84	1.21	-
	A-RSFSCC 100	-	-	-	-	-	-	1.72	0.0062	-	1.22	3.50	1.10	-
	B-VC Plain	-	-	-	-	-	-	3.57	0.0071	-	1.00	7.26	1.00	-
	B-SFVC 50	-	-	-	-	-	-	3.95	0.0095	-	1.34	8.03	1.11	-
	B-SFVC 70	-	-	-	-	-	-	4.08	0.0112	-	1.58	8.30	1.14	-
	B-SFVC 100	-	-	-	-	-	-	3.82	0.0091	-	1.28	7.77	1.07	-
	B-SCC Plain	-	-	-	-	-	-	3.7	0.0075	-	1.00	7.52	1.00	-
	B-SFSCC 50	-	-	-	-	-	-	4.24	0.0119	-	1.58	8.62	1.15	-
	B-SFSCC 70	-	-	-	-	-	-	4.38	0.0138	-	1.83	8.91	1.18	-
	B-SFSCC 100	-	-	-	-	-	-	4.12	0.0113	-	1.50	8.38	1.11	-
	B-RVC Plain	-	-	-	-	-	-	3.25	0.0065	-	1.00	6.61	1.00	-
	B-RSFVC 50	-	-	-	-	-	-	3.72	0.0086	-	1.32	7.56	1.14	-
	B-RSFVC 70	-	-	-	-	-	-	3.84	0.0102	-	1.57	7.81	1.18	-
	B-RSFVC 100	-	-	-	-	-	-	3.64	0.0082	-	1.26	7.40	1.12	-
	B-RSCC Plain	-	-	-	-	-	-	3.48	0.0069	-	1.00	7.08	1.00	-
	B-RSFSCC 50	-	-	-	-	-	-	4.08	0.0109	-	1.58	8.30	1.17	-
	B-RSFSCC 70	-	-	-	-	-	-	4.16	0.0122	-	1.77	8.46	1.20	-
	B-RSFSCC 100	-	-	-	-	-	-	3.78	0.0104	-	1.51	7.69	1.09	-
[11]	TB1-PC	18.4	0.0016	-	1.00	3.27	1.00	18.4	0.0016	-	1.00	3.27	1.00	1.00
	TB2-SFRC25	17.9	0.0015	-	0.91	3.18	0.97	20.3	0.0084	-	5.38	3.61	1.10	1.13
	TB3-SFRC50	17.9	0.0016	-	0.97	3.18	0.97	19.5	0.0021	-	1.32	3.47	1.06	1.09
[12]	M0S	20.4	0.0015	-	1.00	7.36	1.00	25.9	0.0031	-	1.00	9.34	1.00	1.27
	M0.5S	26.3	0.0021	-	1.33	9.49	1.29	43.1	0.0136	-	4.43	15.55	1.66	1.64
	M1S	29.4	0.0022	-	1.40	10.61	1.44	46.7	0.0201	-	6.54	16.85	1.80	1.59
[13]	B05-SF0-T	3.225	-	-	-	3.19	1.00	3.386	-	0.1095	1.00	3.35	1.00	1.05
	B06-SF0.5-T	4.192	-	-	-	4.15	1.30	4.353	-	0.1173	1.07	4.31	1.29	1.04
	B07-SF0.75-T	4.353	-	-	-	4.31	1.35	4.676	-	0.1454	1.33	4.63	1.38	1.07
	B08-SF1-T	4.745	-	-	-	4.70	1.47	5.061	-	0.1894	1.73	5.01	1.49	1.07
[14]	NWC	2.146	-	-	-	6.19	1.00	2.55	0.0624	-	1.00	7.36	1.00	1.19
	NWFRC	3.825	-	-	-	11.04	1.78	4.05	0.084	-	1.35	11.69	1.59	1.06
	CSC	4.35	-	-	-	12.55	1.00	4.8	0.077	-	1.00	13.85	1.00	1.10
	CSFRC	5.1	-	-	-	14.72	1.17	5.325	0.0134	-	0.17	15.37	1.11	1.04
[15]	MIX-1	-	-	-	-	-	-	2.5	-	0.0127	1.00	4.38	1.00	-
	MIX-2	-	-	-	-	-	-	3.072	-	0.0225	1.77	5.38	1.23	-
[16]	NWC	5.49	0.024	-	1.00	5.44	1.00	5.49	0.024	-	1.00	5.44	1.00	1.00
	NWFRC-55	6.95	0.047	-	1.96	6.88	1.27	6.98	0.051	-	2.13	6.91	1.27	1.00
	NWFRC-65	7.1	0.04	-	1.67	7.03	1.29	7.68	0.052	-	2.17	7.60	1.40	1.08
	NWFRC-80	7.8	0.048	-	2.00	7.72	1.42	7.57	0.051	-	2.13	7.49	1.38	0.97
	OPSC-0	5.49	0.052	-	1.00	5.44	1.00	5.5	0.052	-	1.00	5.45	1.00	1.00
	OPSFRC-55	7.28	0.069	-	1.33	7.21	1.33	7.36	0.074	-	1.42	7.29	1.34	1.01
	OPSFRC-65	7.77	0.068	-	1.31	7.69	1.42	7.88	0.077	-	1.48	7.80	1.43	1.01
	OPSFRC-80	8.58	0.076	-	1.46	8.49	1.56	8.6	0.08	-	1.54	8.51	1.56	1.00
[17]	Ap	1.12	0.004	-	1.00	1.59	1.00	1.12	0.004	-	1.00	1.59	1.00	1.00
	A100	2.8	0.0014	-	1.00	3.98	1.00	6.44	0.0063	-	1.00	9.16	1.00	2.30
	A80	3.36	0.0049	-	1.00	4.78	1.00	8.96	0.0411	-	1.00	12.75	1.00	2.67
	Dp	2.38	0.0063	-	1.58	3.39	2.13	2.38	0.0063	-	1.58	3.39	2.13	1.00
	D100	2.92	0.0046	-	3.34	4.16	1.04	11.48	0.0667	-	10.58	16.34	1.78	3.93
	D80	3.36	0.0013	-	0.27	4.78	1.00	14.28	0.0769	-	1.87	20.32	1.59	4.25
	Ep	2.8	0.0035	-	0.88	3.98	2.50	3.08	0.0131	-	3.27	4.38	2.75	1.10
	E100	4.48	0.0035	-	2.56	6.38	1.60	17.36	0.0528	-	8.38	24.71	2.70	3.88
	E80	4.48	0.002	-	0.41	6.38	1.33	18.48	0.0648	-	1.58	26.30	2.06	4.13
[18]	T0	5.27	0.068	-	1.00	5.22	1.00	5.28	0.068	-	1.00	5.23	1.00	1.00
	T25	6.93	0.067	-	0.99	6.86	1.31	6.9	0.072	-	1.06	6.83	1.31	1.00
	T50	7.23	0.078	-	1.15	7.16	1.37	7.32	0.085	-	1.25	7.25	1.39	1.01
	T75	7.73	0.078	-	1.15	7.65	1.47	7.85	0.087	-	1.28	7.77	1.49	1.02
	T100	8.34	0.077	-	1.13	8.26	1.58	8.5	0.089	-	1.31	8.42	1.61	1.02
[19]	TR-00	-	-	-	-	-	-	9.37	0.075	-	1.00	7.05	1.00	-
	TS-15	-	-	-	-	-	-	14.42	0.1045	-	1.39	10.85	1.54	-
[20]	L08F00V0	4.93	0.0032	-	1.00	4.88	1.00	4.93	0.0032	-	1.00	4.88	1.00	1.00
	L08F40V3	4.58	0.0032	-	1.01	4.53	0.93	4.58	0.0032	-	1.01	4.53	0.93	1.00
	L08F40V6	4.62	0.0035	-	1.10	4.57	0.94	5.68	0.0648	-	20.25	5.62	1.15	1.23
	L08F55V3	4.93	0.0038	-	1.19	4.88	1.00	4.94	0.0039	-	1.21	4.89	1.00	1.00
	L08F55V6	5.1	0.004	-	1.23	5.05	1.03	5.87	0.0593	-	18.52	5.81	1.19	1.15
	L08F67V3	4.85	0.0036	-	1.11	4.80	0.98	4.92	0.0056	-	1.76	4.87	1.00	1.01
	L08F67V6	4.91	0.0036	-	1.13	4.86	1.00	5.88	0.0659	-	20.61	5.82	1.19	1.20
	L08F80V3	4.8	0.0034	-	1.05	4.75	0.97	4.85	0.0041	-	1.28	4.80	0.98	1.01
	L08F80V6	4.51	0.0036	-	1.13	4.46	0.91	5.49	0.0634	-	19.82	5.44	1.11	1.22
	L12F00V0	4.45	0.0032	-	1.00	4.41	1.00	5.07	0.0346	-	1.00	5.02	1.00	1.14
	L12F40V3	4.61	0.0037	-	1.16	4.56	1.04	6.01	0.0897	-	2.59	5.95	1.19	1.30
	L12F80V3	4.47	0.0036	-	1.14	4.43	1.00	6.25	0.0935	-	2.70	6.19	1.23	1.40
[21]	R50C-P	2.057	0.039	-	1.00	4.18	1.00	6.584	0.138	-	1.00	13.39	1.00	3.20
	R50C-F1	2.199	0.047	-	1.21	4.47	1.07	6.67	0.141	-	1.02	13.56	1.01	3.03
	R50C-F2	2.367	0.052	-	1.33	4.81	1.15	6.755	0.141	-	1.02	13.74	1.03	2.85
	R50C-F3	2.71	0.057	-	1.46	5.51	1.32	6.841	0.141	-	1.02	13.91	1.04	2.52
	R50C-F4	3.039	0.06	-	1.54	6.18	1.48	6.96	0.141	-	1.02	14.15	1.06	2.29
	R50L-P	2.05	0.039	-	1.00	4.17	1.00	5.131	0.173	-	1.00	10.43	1.00	2.50
	R50L-F1	2.152	0.049	-	1.26	4.38	1.05	5.216	0.173	-	1.00	10.61	1.02	2.42
	R50L-F2	2.467	0.058	-	1.49	5.02	1.20	5.302	0.176	-	1.02	10.78	1.03	2.15
	R50L-F3	2.707	0.063	-	1.62	5.50	1.32	5.387	0.179	-	1.03	10.95	1.05	1.99
	R50L-F4	3.02	0.068	-	1.74	6.14	1.47	5.473	0.18	-	1.04	11.13	1.07	1.81
	R50T-P	2.066	0.034	-	1.00	4.20	1.00	5.687	0.156	-	1.00	11.56	1.00	2.75
	R50T-F1	2.323	0.045	-	1.32	4.72	1.12	5.772	0.156	-	1.00	11.74	1.01	2.48
	R50T-F2	2.602	0.043	-	1.26	5.29	1.26	5.815	0.163	-	1.04	11.82	1.02	2.23
	R50T-F3	2.827	0.053	-	1.56	5.75	1.37	5.9	0.163	-	1.04	12.00	1.04	2.09
	R50T-F4	3.09	0.061	-	1.79	6.28	1.50	5.986	0.167	-	1.07	12.17	1.05	1.94
[22]	RP0	1.45	0.009	-	1.00	2.95	1.00	1.45	0.009	-	1.00	2.95	1.00	1.00
	RP1	1.84	0.019	-	2.11	3.74	1.27	1.84	0.019	-	2.11	3.74	1.27	1.00
	RL0	1.47	0.015	-	1.00	2.99	1.00	1.47	0.015	-	1.00	2.99	1.00	1.00
	RL1	1.8	0.012	-	0.80	3.66	1.22	2.41	0.021	-	1.40	4.90	1.64	1.34
	RR0	1.6	0.015	-	1.00	3.25	1.00	2.31	0.073	-	1.00	4.70	1.00	1.44
	RR1	2	0.016	-	1.07	4.07	1.25	2.73	0.088	-	1.21	5.55	1.18	1.37
[23]	RL-P	1.582	0.009	-	1.00	3.22	1.00	1.582	0.0267	-	1.00	3.22	1.00	1.00
	RL-F1	1.952	0.01	-	1.11	3.97	1.23	2.01	0.0233	-	0.87	4.09	1.27	1.03
	RL-F2	2.254	0.01	-	1.11	4.58	1.42	2.266	0.0267	-	1.00	4.61	1.43	1.01
	RL-F3	2.596	0.011	-	1.22	5.28	1.64	2.608	0.03	-	1.12	5.30	1.65	1.00
	RL-F4	2.791	0.012	-	1.33	5.68	1.76	2.822	0.03	-	1.12	5.74	1.78	1.01
	RT-P	1.546	0.008	-	1.00	3.14	1.00	1.57	0.008	-	1.00	3.19	1.00	1.02
	RT-F1	1.721	0.009	-	1.13	3.50	1.11	1.753	0.0233	-	2.91	3.56	1.12	1.02
	RT-F2	2.29	0.01	-	1.25	4.66	1.48	2.09	0.0267	-	3.34	4.25	1.33	0.91
	RT-F3	2.54	0.012	-	1.50	5.16	1.64	2.566	0.03	-	3.75	5.22	1.63	1.01
	RT-F4	2.664	0.012	-	1.50	5.42	1.72	2.694	0.028	-	3.50	5.48	1.72	1.01
[24]	R40C-P	1.982	-	-	-	4.03	1.00	5.516	-	-	-	11.22	1.00	2.78
	R40C-F1	2.389	-	-	-	4.86	1.21	5.558	-	-	-	11.30	1.01	2.33
	R40C-F2	2.489	-	-	-	5.06	1.26	5.687	-	-	-	11.56	1.03	2.28
	R40C-F3	2.627	-	-	-	5.34	1.33	5.729	-	-	-	11.65	1.04	2.18
	R40C-F4	2.809	-	-	-	5.71	1.42	5.815	-	-	-	11.82	1.05	2.07
	R40L-P	2.036	-	-	-	4.14	1.00	4.062	-	-	-	8.26	1.00	2.00
	R40L-F1	2.189	-	-	-	4.45	1.08	4.105	-	-	-	8.35	1.01	1.88
	R40L-F2	2.277	-	-	-	4.63	1.12	4.19	-	-	-	8.52	1.03	1.84
	R40L-F3	2.449	-	-	-	4.98	1.20	4.233	-	-	-	8.61	1.04	1.73
	R40L-F4	2.795	-	-	-	5.68	1.37	4.233	-	-	-	8.61	1.04	1.51
	R40T-P	2.016	-	-	-	4.10	1.00	3.763	-	-	-	7.65	1.00	1.87
	R40T-F1	2.396	-	-	-	4.87	1.19	3.848	-	-	-	7.82	1.02	1.61
	R40T-F2	2.504	-	-	-	5.09	1.24	3.934	-	-	-	8.00	1.05	1.57
	R40T-F3	2.651	-	-	-	5.39	1.31	3.976	-	-	-	8.08	1.06	1.50
	R40T-F4	2.806	-	-	-	5.71	1.39	4.019	-	-	-	8.17	1.07	1.43
[25]	LC1	-	-	-	-	-	-	0.725	-	-	-	1.47	1.00	-
	LC1-0.5-32	-	-	-	-	-	-	0.772	-	-	-	1.57	1.06	-
	LC1-1.0-32	-	-	-	-	-	-	0.803	-	-	-	1.63	1.11	-
	LC1-1.5-32	-	-	-	-	-	-	0.848	-	-	-	1.72	1.17	-
	LC2	-	-	-	-	-	-	0.867	-	-	-	1.76	1.00	-
	LC2-0.5-25	-	-	-	-	-	-	0.864	-	-	-	1.76	1.00	-
	LC2-1.0-25	-	-	-	-	-	-	0.961	-	-	-	1.95	1.11	-
	LC2-1.5-25	-	-	-	-	-	-	1.102	-	-	-	2.24	1.27	-
	LC2-0.5-32	-	-	-	-	-	-	0.905	-	-	-	1.84	1.04	-
	LC2-1.0-32	-	-	-	-	-	-	1.004	-	-	-	2.04	1.16	-
	LC2-1.5-32	-	-	-	-	-	-	1.16	-	-	-	2.36	1.34	-
	NC	-	-	-	-	-	-	1.429	-	-	-	2.91	1.00	-
	NC-0.5-32	-	-	-	-	-	-	1.461	-	-	-	2.97	1.02	-
	NC-1.0-32	-	-	-	-	-	-	1.604	-	-	-	3.26	1.12	-
	NC-1.5-32	-	-	-	-	-	-	1.818	-	-	-	3.70	1.27	-
	HC	-	-	-	-	-	-	1.91	-	-	-	3.88	1.00	-
	HC-0.5-25	-	-	-	-	-	-	1.925	-	-	-	3.91	1.01	-
	HC-1.0-25	-	-	-	-	-	-	2.108	-	-	-	4.29	1.10	-
	HC-1.5-25	-	-	-	-	-	-	2.282	-	-	-	4.64	1.19	-
	HC-0.5-32	-	-	-	-	-	-	2.032	-	-	-	4.13	1.06	-
	HC-1.0-32	-	-	-	-	-	-	2.317	-	-	-	4.71	1.21	-
	HC-1.5-32	-	-	-	-	-	-	2.732	-	-	-	5.56	1.43	-
	HC-0.5-50	-	-	-	-	-	-	2.098	-	-	-	4.27	1.10	-
	HC-1.0-50	-	-	-	-	-	-	2.65	-	-	-	5.39	1.39	-
[26]	P20-P	-	-	-	-	-	-	1.753	0.0057	-	1.00	3.56	1.00	-
	P20-F1	-	-	-	-	-	-	1.881	0.0059	-	1.05	3.82	1.07	-
	P20-F2	-	-	-	-	-	-	1.924	0.0061	-	1.08	3.91	1.10	-
	P20-F3	-	-	-	-	-	-	2.095	0.0063	-	1.11	4.26	1.20	-
	P20-F4	-	-	-	-	-	-	2.266	0.0064	-	1.14	4.61	1.29	-
	P30-P	-	-	-	-	-	-	1.967	0.0048	-	1.00	4.00	1.00	-
	P30-F1	-	-	-	-	-	-	2.18	0.0059	-	1.24	4.43	1.11	-
	P30-F2	-	-	-	-	-	-	2.351	0.0059	-	1.24	4.78	1.20	-
	P30-F3	-	-	-	-	-	-	2.522	0.0068	-	1.43	5.13	1.28	-
	P30-F4	-	-	-	-	-	-	2.651	0.0077	-	1.62	5.39	1.35	-
	P40-P	-	-	-	-	-	-	2.266	0.0057	-	1.00	4.61	1.00	-
	P40-F1	-	-	-	-	-	-	2.394	0.0066	-	1.16	4.87	1.06	-
	P40-F2	-	-	-	-	-	-	2.522	0.0067	-	1.18	5.13	1.11	-
	P40-F3	-	-	-	-	-	-	2.608	0.0088	-	1.55	5.30	1.15	-
	P40-F4	-	-	-	-	-	-	2.907	0.0093	-	1.65	5.91	1.28	-
	P50-P	-	-	-	-	-	-	2.138	0.0044	-	1.00	4.35	1.00	-
	P50-F1	-	-	-	-	-	-	2.309	0.0067	-	1.51	4.69	1.08	-
	P50-F2	-	-	-	-	-	-	2.651	0.0083	-	1.88	5.39	1.24	-
	P50-F3	-	-	-	-	-	-	2.95	0.0086	-	1.95	6.00	1.38	-
	P50-F4	-	-	-	-	-	-	2.993	0.0086	-	1.96	6.09	1.40	-
[27]	B1	0.966	-	-	-	1.96	1.00	1.077	-	-	-	2.19	1.00	1.11
	B2	1.033	-	-	-	2.10	1.07	1.408	-	-	-	2.86	1.31	1.36
	B3	1.187	-	-	-	2.41	1.23	1.739	-	-	-	3.54	1.61	1.47
	B4	1.297	-	-	-	2.64	1.00	1.712	-	-	-	3.48	1.00	1.32
	B5	1.408	-	-	-	2.86	1.09	2.29	-	-	-	4.66	1.34	1.63
	B6	1.518	-	-	-	3.09	1.17	2.842	-	-	-	5.78	1.66	1.87
[28]	B0.0-0	-	-	-	-	-	-	2.68	-	-	-	4.16	1.00	-
	B0.5-0	-	-	-	-	-	-	2.99	-	-	-	4.64	1.12	-
	B1.0-0	-	-	-	-	-	-	2.8	-	-	-	4.35	1.04	-
	B1.5-0	-	-	-	-	-	-	3.56	-	-	-	5.53	1.33	-
	B0.0-2a	-	-	-	-	-	-	4.44	-	-	-	6.89	1.00	-
	B0.0-2b	-	-	-	-	-	-	4.89	-	-	-	7.59	1.00	-
	B0.5-2	-	-	-	-	-	-	4.81	-	-	-	7.47	1.03	-
	B1.0-2	-	-	-	-	-	-	5.09	-	-	-	7.90	1.05	-
	B1.5-2	-	-	-	-	-	-	6.19	-	-	-	9.61	1.25	-
	B0.0-4a	-	-	-	-	-	-	4.57	-	-	-	7.10	1.00	-
	B0.0-4b	-	-	-	-	-	-	5.58	-	-	-	8.66	1.00	-
	B0.5-4	-	-	-	-	-	-	5.36	-	-	-	8.32	1.06	-
	B1.0-4	-	-	-	-	-	-	5.32	-	-	-	8.26	1.05	-
	B1.5-4	-	-	-	-	-	-	6.25	-	-	-	9.70	1.23	-
[29]	P1	0.158	-	-	-	0.09	1.00	0.158	-	-	-	0.09	1.00	1.00
	P2	0.17	-	-	-	0.10	1.08	0.17	-	-	-	0.10	1.08	1.00
	P3	0.158	-	-	-	0.09	1.00	0.158	-	-	-	0.09	1.00	1.00
	P4	0.155	-	-	-	0.09	0.98	0.155	-	-	-	0.09	0.98	1.00
[30]	1	-	-	-	-	-	-	0.8437	-	-	-	3.82	1.00	-
	2	-	-	-	-	-	-	0.8142	-	-	-	3.69	1.00	-
	3	-	-	-	-	-	-	0.824	-	-	-	3.73	1.00	-
	4	-	-	-	-	-	-	0.9048	-	-	-	4.10	1.09	-
	5	-	-	-	-	-	-	0.9025	-	-	-	4.08	1.09	-
	6	-	-	-	-	-	-	0.8927	-	-	-	4.04	1.08	-
	7	-	-	-	-	-	-	0.9228	-	-	-	4.18	1.12	-
	8	-	-	-	-	-	-	0.922	-	-	-	4.17	1.11	-
	9	-	-	-	-	-	-	0.9152	-	-	-	4.14	1.11	-
	10	-	-	-	-	-	-	0.9221	-	-	-	4.17	1.11	-
	11	-	-	-	-	-	-	0.9633	-	-	-	4.36	1.16	-
	12	-	-	-	-	-	-	0.9025	-	-	-	4.08	1.09	-
	13	-	-	-	-	-	-	0.9957	-	-	-	4.51	1.20	-
	14	-	-	-	-	-	-	0.9319	-	-	-	4.22	1.13	-
	15	-	-	-	-	-	-	0.9593	-	-	-	4.34	1.16	-
[31]	AP	-	-	-	-	-	-	1.1	-	-	-	5.28	1.00	-
	BP	-	-	-	-	-	-	1.084	-	-	-	5.21	1.00	-
	CP	-	-	-	-	-	-	0.98	-	-	-	4.71	1.00	-
	AF	-	-	-	-	-	-	1.365	-	-	-	6.56	1.24	-
	BF	-	-	-	-	-	-	1.21	-	-	-	5.81	1.12	-
	CF	-	-	-	-	-	-	0.94	-	-	-	4.51	0.96	-
[32]	B1	12	-	-	-	7.23	1.00	14.112	0.0122	-	1.00	8.50	1.00	1.18
	B4	13.05	-	-	-	7.86	1.09	21.672	0.0311	-	2.55	13.06	1.54	1.66
[33]	B-NC	1	-	0.002	1.00	2.89	1.00	3.4	-	0.0204	1.00	9.81	1.00	3.40
	B-SSF-13	1.2	-	0.002	1.00	3.46	1.20	4.2	-	0.0384	1.88	12.12	1.24	3.50
	B-HSF-13	1.1	-	0.0018	0.90	3.17	1.10	3.9	-	0.0035	0.17	11.26	1.15	3.55

*T_cr_*—cracking moment, *θ_cr_*—angle at the cracking moment, *θ_cr_^0^*—angle at the cracking moment for reference beams, *τ_cr_*—shear stresses of the cross-section for the cracking moment, *τ_cr_^0^*—shear stresses of the cross-section for the cracking moment of reference beams, *T_u_*—maximum moment, *θ_u_*—angle at the maximum moment, *θ_u_^0^*—angle at the maximum moment for reference beams, *τ_u_*—shear stresses of the cross-section for the maximum moment, *τ_u_^0^*—shear stresses of the cross-section for the maximum moment of reference beams.
